# Development of physiologically‐based pharmacokinetic models for standard of care and newer tuberculosis drugs

**DOI:** 10.1002/psp4.12707

**Published:** 2021-10-08

**Authors:** Helen Humphries, Lisa Almond, Alexander Berg, Iain Gardner, Oliver Hatley, Xian Pan, Ben Small, Mian Zhang, Masoud Jamei, Klaus Romero

**Affiliations:** ^1^ Certara UK Limited, Simcyp Division Sheffield UK; ^2^ Critical Path Institute Tucson Arizona USA

## Abstract

Tuberculosis (TB) remains a global health problem and there is an ongoing effort to develop more effective therapies and new combination regimes that can reduce duration of treatment. The purpose of this study was to demonstrate utility of a physiologically‐based pharmacokinetic modeling approach to predict plasma and lung concentrations of 11 compounds used or under development as TB therapies (bedaquiline [and *N*‐desmethyl bedaquiline], clofazimine, cycloserine, ethambutol, ethionamide, isoniazid, kanamycin, linezolid, pyrazinamide, rifampicin, and rifapentine). Model accuracy was assessed by comparison of simulated plasma pharmacokinetic parameters with healthy volunteer data for compounds administered alone or in combination. Eighty‐four percent (area under the curve [AUC]) and 91% (maximum concentration [*C*_max_]) of simulated mean values were within 1.5‐fold of the observed data and the simulated drug‐drug interaction ratios were within 1.5‐fold (AUC) and twofold (*C*_max_) of the observed data for nine (AUC) and eight (*C*_max_) of the 10 cases. Following satisfactory recovery of plasma concentrations in healthy volunteers, model accuracy was assessed further (where patients’ with TB data were available) by comparing clinical data with simulated lung concentrations (9 compounds) and simulated lung: plasma concentration ratios (7 compounds). The 5th–95th percentiles for the simulated lung concentration data recovered between 13% (isoniazid and pyrazinamide) and 88% (pyrazinamide) of the observed data points (*Am J Respir Crit Care Med*, 198, 2018, 1208; *Nat Med*, 21, 2015, 1223; *PLoS Med*, 16, 2019, e1002773). The impact of uncertain model parameters, such as the fraction of drug unbound in lung tissue mass (fu_mass_), is discussed. Additionally, the variability associated with the patient lung concentration data, which was sparse and included extensive within‐subject, interlaboratory, and experimental variability (as well interindividual variability) is reviewed. All presented models are transparently documented and are available as open‐source to aid further research.

**Study Highlights**

**WHAT IS THE CURRENT KNOWLEDGE ON THE TOPIC?**

Physiologically‐based pharmacokinetic (PBPK) models have been described in the literature for selected anti‐tuberculosis (TB) compounds, but the ability of PBPK models to simulate the plasma and lung concentrations of anti‐TB agents has not been reported for many of the drugs used to treat TB, including newer agents.

**WHAT QUESTION DID THIS STUDY ADDRESS?**

Can PBPK models that describe plasma and lung concentrations of anti‐TB agents be developed? Can the PBPK models be modified to account for physiology and demographic differences in patients with TB compared with healthy subjects?

**WHAT DOES THIS STUDY ADD TO OUR KNOWLEDGE** **?**

PBPK models were developed for 11 standard‐of‐care and newer anti‐TB agents and the plasma and lung concentrations predicted by the models were compared with available clinical data.

**HOW MIGHT THIS CHANGE DRUG DISCOVERY, DEVELOPMENT, AND/OR THERAPEUTICS?**

The PBPK models have the potential to help with the design of clinical studies to optimize dosing regimens of TB therapy.

## INTRODUCTION

Despite efforts to improve the available treatments for tuberculosis (TB), this infection remains a global health burden with an estimated 1.6 million deaths recorded in 2017.^1^ A cornerstone of current TB therapy is the use of combinations of drugs because no single agent proves to be as effective as monotherapy.^2^ Current regimens for drug‐susceptible TB require treatment with up to four co‐administered drugs and a long treatment duration (i.e., 6 months). In the context of drug‐resistant strains, the treatment duration and pill burden become even more challenging. To improve the effective options for patients and to curb the global pandemic, efforts to develop new TB treatments are ongoing. Bedaquiline was approved by the US Food and Drug Administration (FDA) in 2012 and several other novel agents are at various stages of the drug development process.^3^ It is anticipated that emerging drugs for TB will be combined into shorter duration regimens, whereas remaining highly effective for both drug susceptible and drug‐resistant strains. However, given the large size of the development pipeline for new agents, efficiently evaluating the vast number of potential drug combinations is a challenging task.^3^ Approaches that enable the selection of the most promising combinations early in the drug development process are essential.

In silico modeling and simulation (M&S) approaches provide a mechanism for understanding experimental drug data as it is generated and also for guiding the decision making procedure in drug and regimen development programs.^4^ Mechanistic M&S approaches have the potential to be particularly amenable to inform drug combination selection in TB, as they can link exposure and response data for new compounds to the existing knowledge of more widely studied drugs in order to predict drug pharmacodynamic (PD) effects.^5^, ^6^ One such mechanistic modeling approach utilizes physiologically‐based pharmacokinetic (PBPK) models. PBPK models combine information on human physiology, demographics, and pharmacogenetics, together with information on drug properties (physicochemical, binding, permeability, metabolism, and, if appropriate, transport) into a computational model that allows the plasma and tissue concentrations of the compound to be simulated.^4^ The predicted concentrations in the target tissues can then be compared to known or expected concentration‐related targets for efficacy and/or safety or even linked to response measurements in the form of PBPK/PD models.^6^ Such PBPK and PBPK/PD models, typically developed initially for single‐agents, have the potential to be extended to assess the pharmacokinetic (PK) drug interactions and synergistic/antagonistic PD effects of co‐administered drugs simultaneously, which is important for the TB regimen development process, given the strong reliance on combination therapy.

To facilitate the application of mechanistic PBPK modeling in TB drug development, PBPK models were constructed for 11 standard‐of‐care and experimental TB drugs. These drugs include bedaquiline (and *N*‐desmethyl bedaquiline), clofazimine, cycloserine, ethambutol, ethionamide, isoniazid, kanamycin, linezolid, pyrazinamide, rifampicin, and rifapentine, as well as moxifloxacin, which will be detailed in a separate publication. In addition, to support model verification and application in a relevant patient population, data were gathered on the major physiological changes (body weight, plasma protein levels, etc.) that occur during TB infection. These were used to construct a virtual population of South African patients infected with TB (detailed in Supplementary Material). The drug PBPK models and virtual TB population are available for research purposes (https://members.simcyp.com/account/globalHealthRepository). This manuscript details the development of these PBPK models, which were constructed using a previously published multicompartment permeability‐limited lung model^7^ (Figure S1) and compared with available plasma, and, where possible, lung and drug concentration data to assess model performance.

## METHODS

**FIGURE 1:**
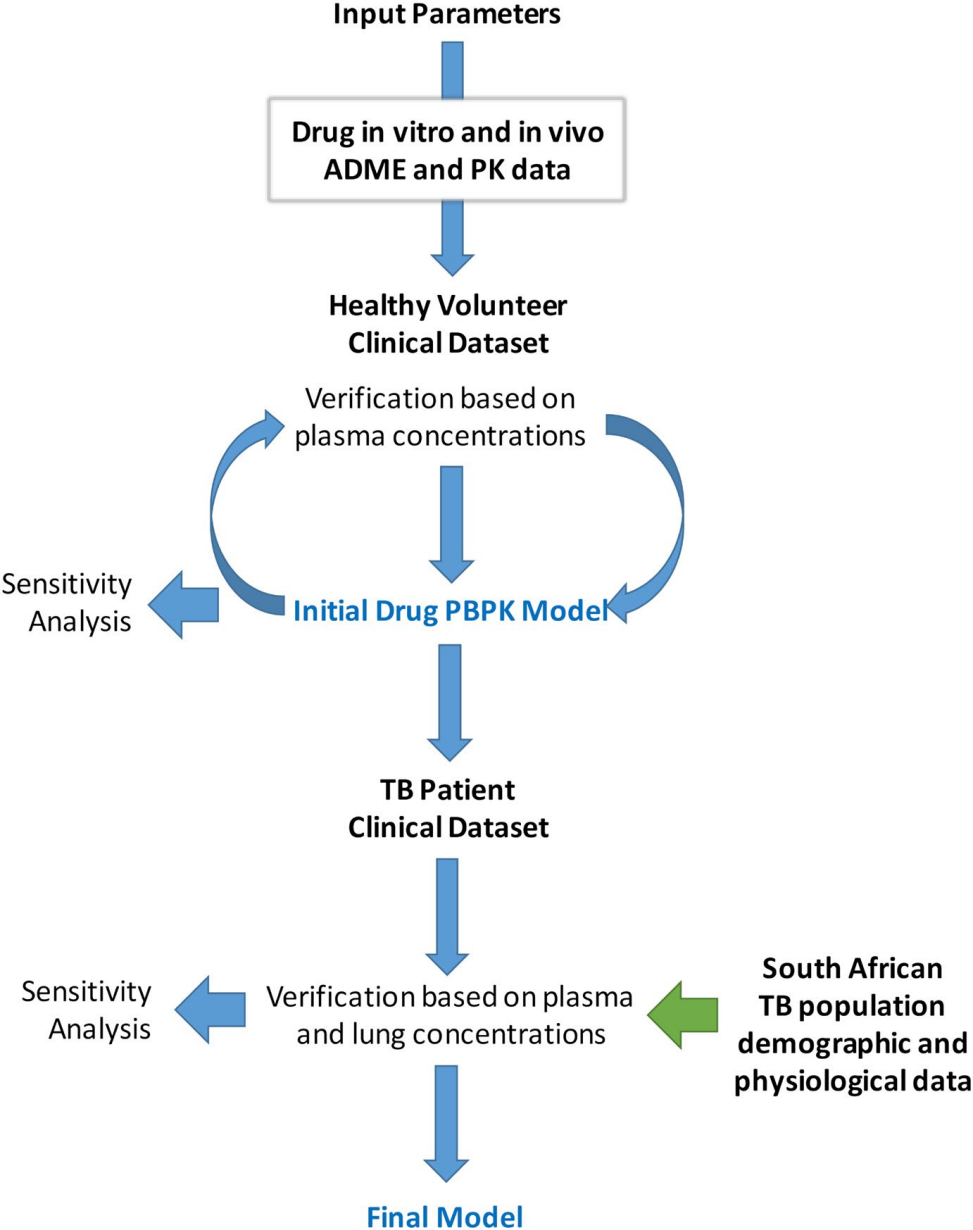
Workflow outlining the process used to construct PBPK models for the 13 anti‐TB compounds. ADME, absorption, distribution, metabolism, and excretion; PBPK, physiologically‐based pharmacokinetic; PK, pharmacokinetic; TB, tuberculosis

PBPK models were constructed in the Simcyp Simulator (V16 release 1) using the workflow illustrated in Figure 1. The performance of the PBPK models was compared against clinical data. Ten simulated trials were conducted with virtual subjects that matched (by the number, age range, and proportion of women) the subjects used in each clinical study (the simulation design is described in Table S3). A base model was constructed for each compound using in vitro or in silico data (plasma protein binding, blood to plasma ratio, lipophilicity, pKa, rate of metabolism in human liver microsomes, hepatocytes, or human recombinant enzymes, etc.). A workaround for a known software issue was included in the simulations to ensure blood pH (7.4) was used to calculate unionized drug concentration in the pulmonary blood compartment (https://members.simcyp.com—corrected in the more recent Simulator version 20).

If necessary, the model was refined to ensure that the model closely reflected the clinical data. The performance of the final model was then checked (where possible) against independent clinical data not used in the model refinement. For rifampicin, rifapentine, and bedaquiline (selected due to availability of relevant clinical data with sensitive substrates and/or strong inhibitors of specific CYP enzymes), drug interactions were simulated. Simulations investigating the change in clearance of midazolam when co‐dosed with rifapentine at steady‐state, were conducted at doses of 5, 10, 15, and 20 mg/kg using a study design analogous to that used in the clinical study by Dooley et al.^8^ A list of the different clinical studies that were simulated and the details of the populations used for each simulation are provided in Table S3.

### Model assumptions

The PBPK models were constructed using full body PBPK models where different organs of the body are represented as compartments with specified blood flow, volume, and tissue composition. The disposition of the compounds into the lung was modeled using a permeability‐limited lung model that has been previously described in detail^7^ (Figure S1). The other organs were generally assumed to behave as well‐stirred compartments where distribution of the compound into the tissue is governed by standard perfusion rate‐limited PBPK model equations.^9^ Absorption was described by using either the first order absorption model (with intestinal metabolism described using the Q_gut_ model^10^) or the more complex, regionally distributed permeability‐limited (advanced dissolution, absorption, and metabolism [ADAM]) model, as indicated in Table S1.^11^ Tissue:plasma partition ratios (*K*p) and the extent of tissue binding in the lung (fraction of drug unbound in lung tissue mass [fu_mass_]) were initially predicted using the approach outlined by Rodgers and Rowland and co‐workers.^12^, ^13^, ^14^

**FIGURE 2:**
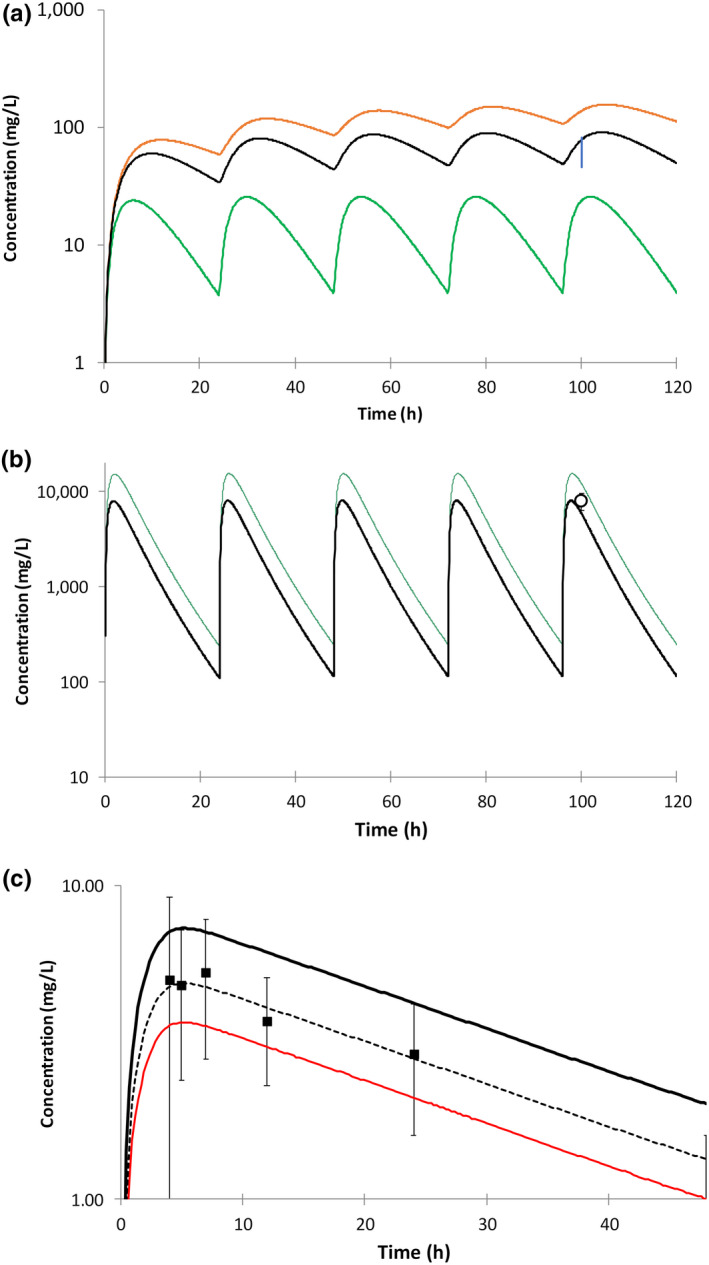
Impact of fu_mass_ on simulated lung concentrations of ethambutol, rifampicin and rifapentine. (a) Impact of fu_mass_ on simulated lung concentration of ethambutol after multiple oral doses of 15 mg/kg. Green line: initial lung model (fu_mass_ = 0.36), orange line: fu_mass_ = 0.036, black line: fu_mass_ = 0.072, vertical blue bar: range of mean ethambutol concentrations in alveolar cells, collected from different subgroups of subjects in the clinical study reported by Conte et al.^22^ (b) Impact of fu_mass_ on simulated lung concentration of rifampicin after multiple oral doses of 600 mg. Green line: initial PBPK model fu_mass_ = 0.058, black line: fu_mass_ increased by a factor of 2 = 0.116, Square symbol: observed data reported by Ziglam et al.^19^ (mean ± SD). (c) Impact of fu_mass_ on simulated lung concentration of rifapentine after a single oral dose of 600 mg. Black line: initial PBPK model fu_mass_ = 0.02, red line: fu_mass_ increased by a factor of 2 = 0.04, dashed black line: fu_mass_ increased by a factor of 1.5 = 0.03, Square symbol: observed data reported by Conte et al.^24^ (mean ± SD). fu_mass_, fraction of drug unbound in lung tissue mass; PBPK, physiologically‐based pharmacokinetic

Final input parameters are listed in Tables S1 and S2 and sources referenced and categorized as experimental, predicted, or optimized. It is important to note a degree of uncertainty associated with some input parameters, particularly those that have been predicted or optimized. Lung fu_mass_ was identified as a particularly sensitive and uncertain parameter and so sensitivity analysis for this parameter is described in the Supplementary Material (Results) and Figure 2. Other compound‐specific assumptions are also described in the Supplementary Material (Methods), including approaches to account for known population variability in *N*‐acetyltransferase 2 metabolism (isoniazid), optimization of fraction metabolized by CYP3A4 (bedaquiline), scaling of flavin‐containing monooxygenase isoform 3 intrinsic clearance (ethionamide), and induction of CYP3A4 (rifapentine).

### Simulation design

Virtual populations used in the simulations are described in Table S3.

#### Development of a population with the physiological characteristics of TB‐infected individuals

**FIGURE 3:**
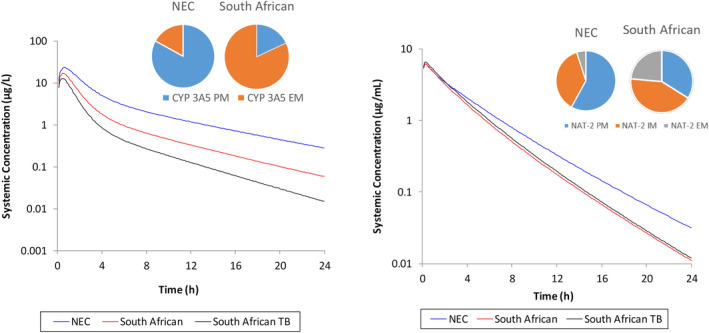
Simulated impact of using either Sim‐North European Caucasian (NEC) or virtual Black South African TB population on midazolam (CYP 3A5) or isoniazid (NAT‐2) exposure. Simulations were run as 10 trials of 10 individuals, 20–50 years, 50% women. Pie charts show the different phenotype frequencies used in the two populations for CYP 3A5 or NAT‐2 extensive metabolizers (EM), intermediate metabolizers (IM), or poor metabolizers (PM; South African population described in more detail in Supplementary Material. Sim‐NEC population used as V16 library file with added NAT‐2 phenotype data published by Sabbagh et al.^26^)

In order to simulate the pharmacokinetics (PKs) of compounds in TB‐infected subjects, a virtual population was constructed to represent a Black South African population. Details of this virtual population are fully described in the Supplementary Material, including data sources for age distribution, relationships between age, height and weight, phenotypic differences for enzyme activity and population frequency, and sources of other physiological data. The impact of changing the population from the default Simcyp library Sim‐North European Caucasian (NEC) population to the South African and South African‐TB infected population for the simulated exposure of midazolam and isoniazid is shown in Figure 3.

### Sensitivity analyses

The sensitivity of the simulated concentration profiles in the plasma and lung to some of the uncertain or sensitive model input parameters was evaluated using local sensitivity analysis. The parameters of interest were adjusted over a range of values that spanned the initial input parameter and the change in simulated output was evaluated. Ranges of parameter values were determined with the aim of improving prediction accuracy and so multiples of initial values used differed for the different compounds. The fu_mass_ values were decreased by 10‐fold (ethambutol) or increased by twofold (rifampicin) or threefold (rifapentine and linezolid) and optimal values selected from within these ranges. The results from these local sensitivity analyses are shown in Figure 2 and in the Supplementary Material (Results section including Table S5).

## RESULTS

### Simulations of drug concentrations in the plasma of healthy volunteers

**FIGURE 4:**
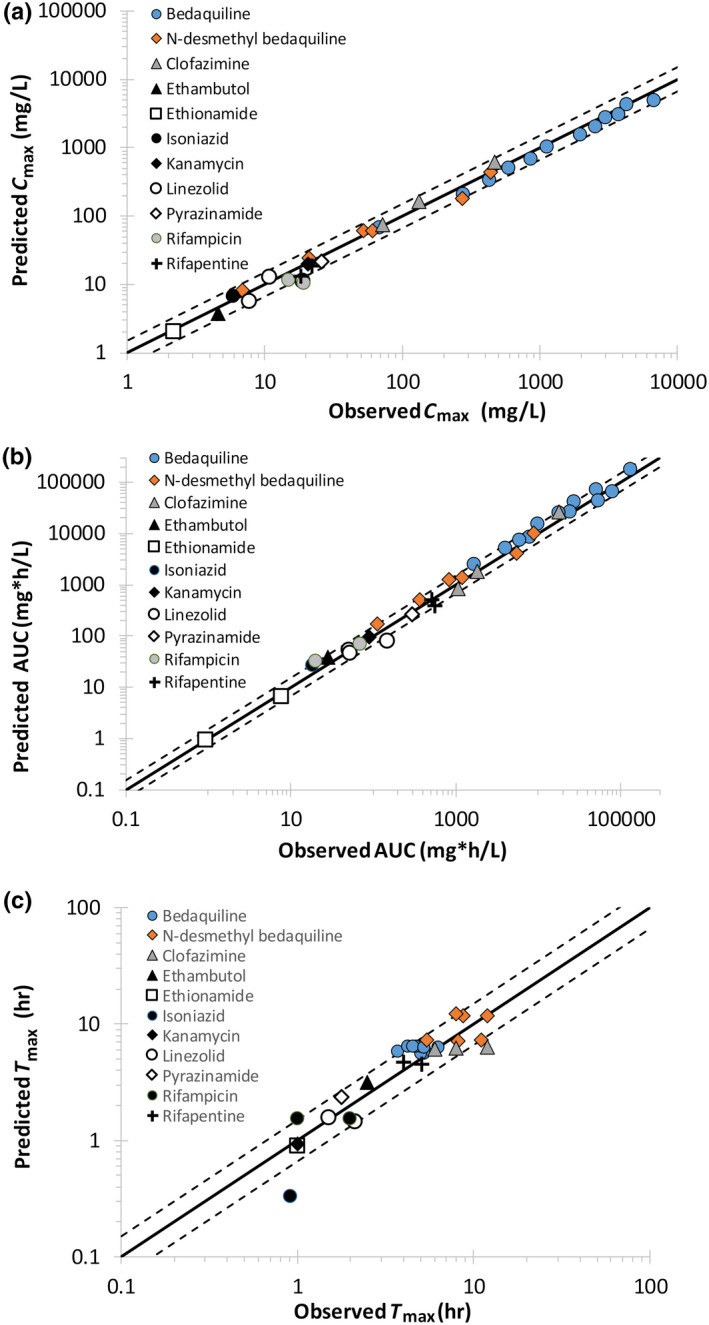
Simulated plasma *C*_max_ (a), AUC (b), and *T*_max_ values (c) in comparison to observed clinical values for 13 tuberculosis (TB) drugs. Solid lines represent unity and dashed lines represent a 1.5‐fold difference in simulated values in comparison to observed. AUC, area under the curve; C_max_, maximum concentration; T_max_, time to maximum concentration

Input parameters used in the PBPK models are listed in Tables S1 and S2. The PBPK models reasonably described the observed plasma maximum concentration (*C*_max_), time to C_max_ (*T*_max_), and area under the curve (AUC) values for a healthy volunteer in the North European population (Figure 4, Figure S2 and Table S4). Ninety‐one percent of simulated *C*_max_, 81% of simulated *T*_max_, and 84% of simulated AUC values were within 1.5‐fold of the observed data. The remaining simulated *C*_max_, *T*_max_, and AUC values were within twofold of the observed values, apart from simulated isoniazid *T*_max_ (Figure 4).

### Simulations of drug–drug interactions in healthy volunteers

For rifapentine, rifampicin, and bedaquiline, drug‐drug interaction (DDI) studies were simulated, and the results are summarized in Table 1. Simulated values for AUC ratio were within 1.5‐fold of the observed values for the effect of rifampicin or rifapentine (5–20 mg/kg) on sensitive CYP 3A4/5 marker substrates (midazolam or triazolam). Simulated values for *C*_max_ ratio were within twofold for the same simulations, except for the effect of 10 mg/kg rifapentine on midazolam (predicted *C*_max_ ratio was 2.5‐fold higher than observed).

**TABLE 1:** Comparison of predicted and observed extent of drug‐drug interaction in healthy volunteers

TB drug (perpetrator)	Substrate	AUC ratio			*C*_max_ ratio			Reference
		Observed	Predicted	Pred/Obs	Observed	Predicted	Pred/Obs	
Rifampicin	Midazolam^d^	0.05	0.07	1.40	0.11	0.10	0.91	43
Rifampicin	Triazolam^d^	0.06	0.07	1.26	0.12	0.12	0.94	44
Rifapentine 5 mg/kg	Midazolam^c^	0.09	0.08	0.88	0.18	0.10	0.55	8
Rifapentine 10 mg/kg	Midazolam^c^	0.09	0.06	0.71	0.20	0.08	0.39	8
Rifapentine 15 mg/kg	Midazolam^c^	0.07	0.06	0.84	0.14	0.07	0.52	8
Rifapentine 20 mg/kg	Midazolam^c^	0.07	0.05	0.80	0.11	0.07	0.64	8
Bedaquiline	Ketoconazole^a^, ^d^	1.22	1.19	0.98	1.09	1.20	1.11	45
*N*‐desmethyl bedaquiline	Ketoconazole^a^, ^d^	1.01	0.69	0.68	1.01	0.69	0.69	45
Bedaquiline	Rifampicin^b^, ^e^	0.48	0.39	0.81	0.57	0.47	0.82	46
*N*‐desmethyl bedaquiline	Rifampicin^b^, ^e^	1.21	3.10	2.82	1.31	3.22	2.46	46

Abbreviations: AUC, area under the curve; C_max_, maximum concentration; Obs, observed; Pred, predicted; TB, tuberculosis.

^a^ Values are arithmetic mean.

^b^ Values are median.

^c^ Used in model development.

^d^ Used for model verification.

^e^ Values are geometric mean.

The impact of the strong CYP 3A4 inhibitor ketoconazole, or CYP 3A inducer rifampicin on bedaquiline were simulated with acceptable accuracy (predicted/observed DDI ratios were between 0.8 and 1.25). The simulated changes in exposure to the *N*‐desmethyl metabolite of bedaquiline were within 0.7 and 2.8‐fold of the observed AUC ratio when co‐dosed with ketoconazole and rifampicin, respectively.

### Observed lung concentration data in patients with TB

**FIGURE 5:**
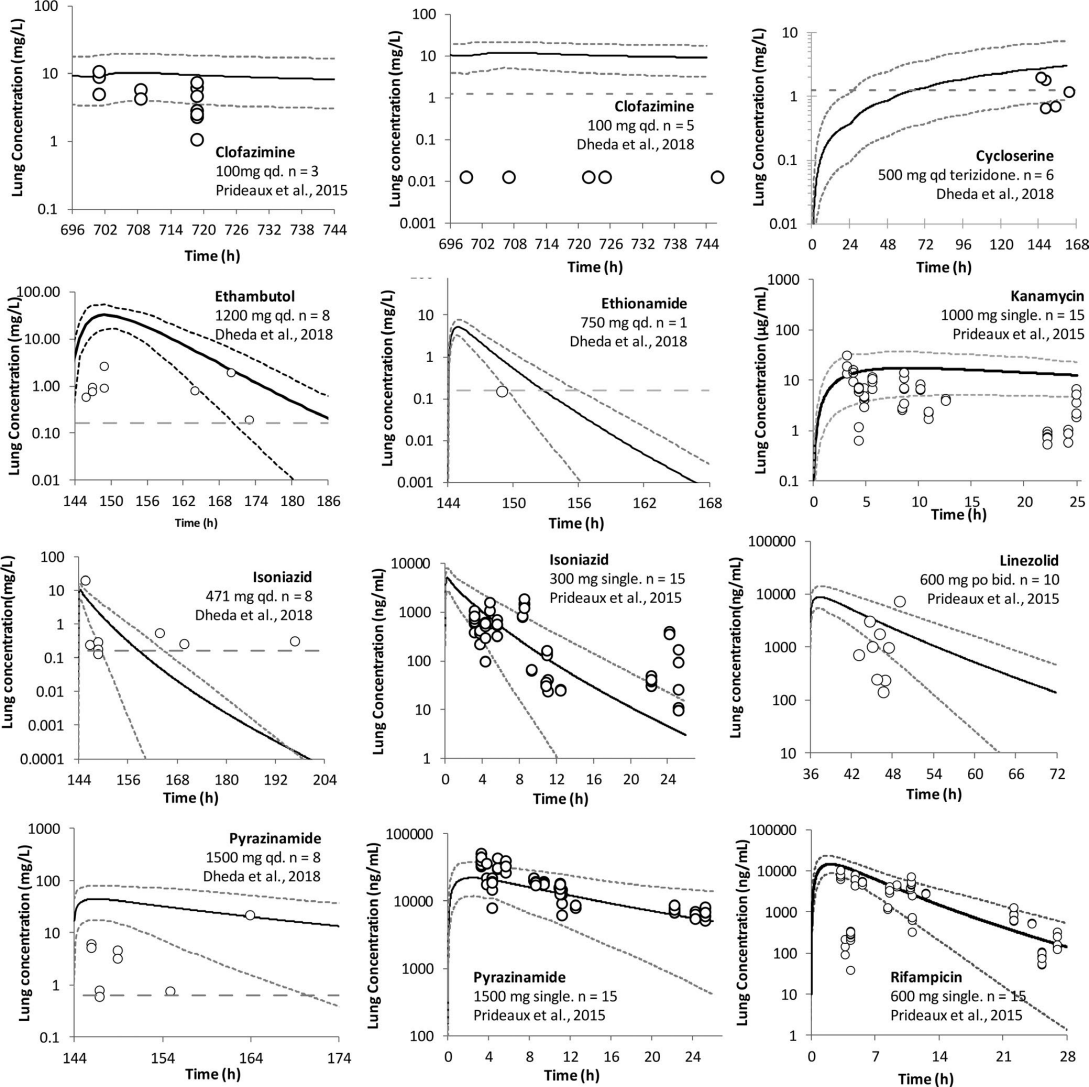
Simulated versus observed concentration in lung tissue mass over time for nine drugs administered to patients with tuberculosis (TB). Simulated (lines) and observed (data points) lung concentration over time for clofazimine, cycloserine, ethambutol, ethionamide, kanamycin, isoniazid, linezolid, pyrazinamide, and rifampicin. The observed data were obtained from Prideaux et al.^15^ (linezolid and kanamycin published by Strydom et al.^16^) and Dheda et al.^17^ The *n* represents the number of patients with TB. Simulated data are from the right lung (RL) compartment and are after the final dose at steady state, except where indicated as single dose (kanamycin, isoniazid, pyrazinamide, and rifampicin). The short dashed lines represent the 5th and 95th percentile of the prediction in the total virtual population (10 trials of 15 healthy volunteer population, 23–59 years, 33% women for the Prideaux et al.^15^ study; 10 trials of 12 Black South African TB population, 23–50 years old, 67% women for the Dheda et al.^17^ study)

Observed lung concentration data in patients with TB were predominantly drawn from two clinical studies reported by Prideaux et al.^15^, ^16^ and Dheda et al.,^17^ which include patients assessed in South Korea and Cape Town, South Africa, respectively. Significant variability was observed for lung concentrations in (1) different patients at different time points postdose^15^, ^16^, ^17^ and also in (2) repeated measurements in the same subject (e.g., range in coefficient of variation [CV] for linezolid 6–67% for 2–7 samples from each of nine patients^15^, ^16^; Figure 5). In addition, 13% (isoniazid and pyrazinamide), 67% (cycloserine), and 100% (clofazimine and ethionamide), of the reported lung concentrations were below the reported lower limit of quantification (LLOQ) of the assay used by Dheda et al.^17^ (Figure 5). This observation needs to be considered when comparing the simulated and observed data. Data points were excluded by Prideaux et al.,^15^, ^16^ if they were below the LLOQ. In some cases (ethambutol and ethionamide), plasma and lung concentrations were not available at the same time postdose and so it was not possible to estimate observed lung‐to‐plasma concentration ratios.^17^

### Simulations of drug distribution to the lung tissue mass of patients with TB

**FIGURE 6:**
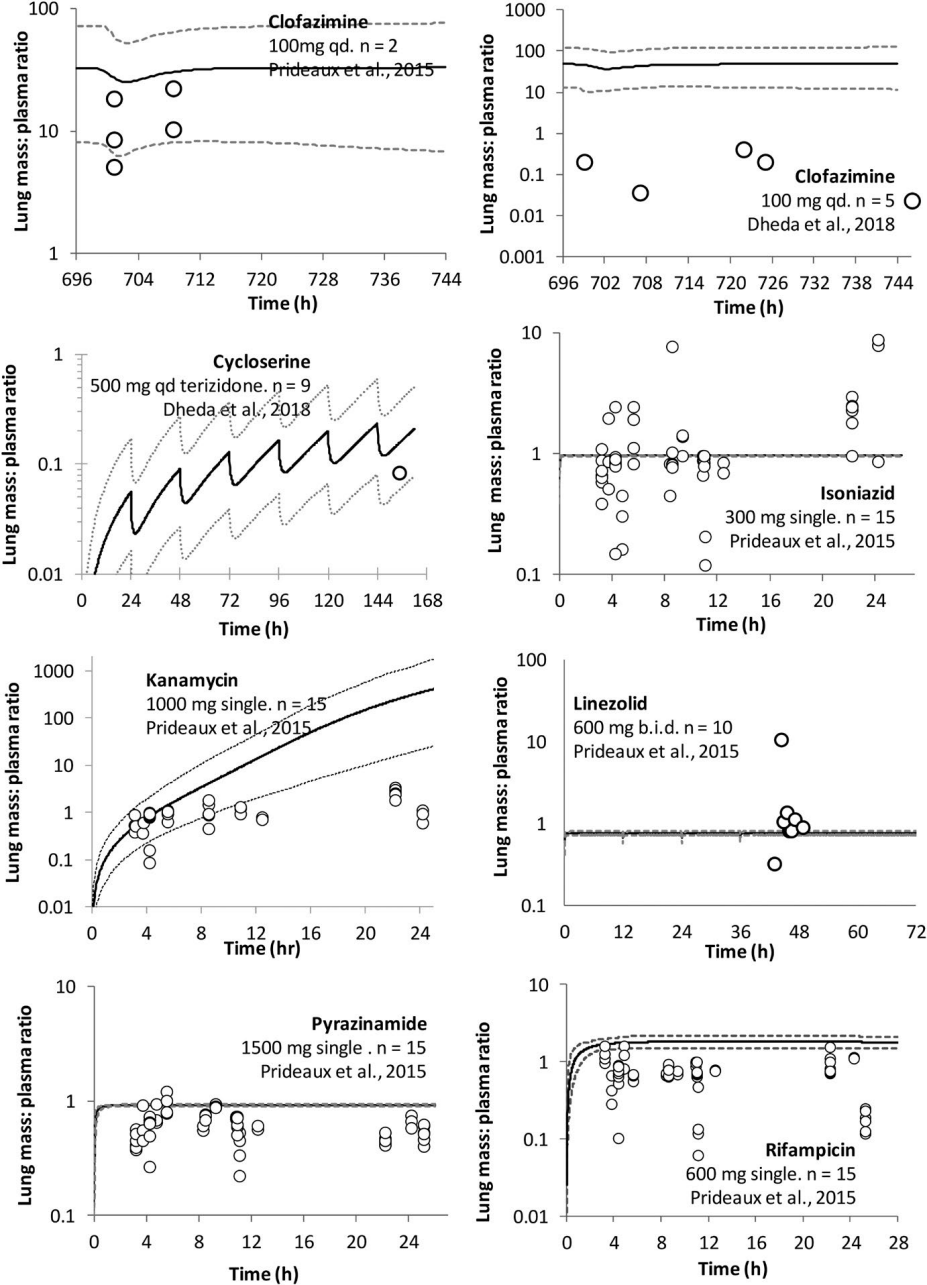
Simulated versus observed lung tissue mass:plasma concentration ratio over time for seven compounds administered to patients with tuberculosis (TB). Simulated (lines) and observed (data points) data from Prideaux et al.^15^ (linezolid and kanamycin data are unpublished) and Dheda et al.^17^ The *n* represents the number of patients. The short dashed lines represent the 5th and 95th percentile of the total virtual population of 10 trials of 15 healthy volunteers, 23–50 years, 33% women,^15^ or 10 trials of 12 individuals, 23–50 years old, 67% women.^17^ Simulated data are for a single oral dose (isoniazid, kanamycin, pyrazinamide, and rifampicin) or after the final dose at steady‐state (cycloserine and clofazimine) and are for the right lung (RL) compartment

Observed drug plasma exposure was variable but generally within the range of the simulations (Figure S3). A comparison of PBPK predicted lung tissue mass concentrations over time against observed lung concentration data is given in Figure 5 for nine compounds where observed data were available (clofazimine, cycloserine, ethambutol, ethionamide, kanamycin, isoniazid, linezolid, pyrazinamide, and rifampicin). Lung concentration data were also analyzed relative to plasma concentration at the same time points postdose in the same patients (Figure 6).

Despite the described issues with observed data assay sensitivity and variability, simulated lung tissue concentrations (lung tissue mass compartment) using an initial PBPK model were reasonably consistent with data reported by either Prideaux et al.^15^, ^16^ or Dheda et al.^17^ (Figure 5) for eight of the nine drugs. The 5th–95th percentiles of the predicted lung concentrations were able to recover 67% (clofazimine), 54% (kanamycin), 81% (isoniazid), 33% (linezolid), 88% (pyrazinamide), and 66% (rifampicin) of the observed data points reported by Prideaux et al.^15^, ^16^ (Figure 5) and 60% (cycloserine), 25% (ethambutol), and 13% (isoniazid and pyrazinamide) of the observed data points reported by Dheda et al.^17^ For clofazimine, cycloserine, and ethionamide, all observed data points reported by Dheda et al. and not captured by the 5th–95th percentiles were below the assay LLOQ^17^ (Figure 5). Observed plasma and lung concentrations were determined by Dheda et al.,^17^ the 20 h post‐pyrazinamide dose were successfully recovered by the predicted 5th–95th percentile concentration range, whereas lung concentrations at initial time points were overpredicted (Figure 5 and Figure S3).

Cycloserine lung concentrations were initially overpredicted using the preliminary PBPK model (6–28 fold).^17^, ^18^, ^19^ However, sensitivity analysis showed that adjusting lung permeability improved model prediction accuracy. For the final model, cycloserine lung permeability was reduced to the lower limit allowed by the software (0.001 10^−4^ cm/s; overprediction reduced to 1.4–4.5‐fold; Figures 5 and 2c).

For cases where observed plasma concentrations were available at the same time postdose as lung concentrations, lung‐to‐plasma concentration ratios were compared with simulated data (Figure 6). For clofazimine, cycloserine, and kanamycin, the 5th–95th percentiles of the predicted lung‐to‐plasma concentration ratios were able to recover 80%, 100%, and 52% of the observed data, respectively. Despite the large amount of variability in the clinical lung concentrations, predicted mean lung‐to‐plasma concentration ratios for isoniazid and pyrazinamide (0.97 and 0.59, respectively) were in good agreement with the observed mean ratios (1.20 and 0.59, respectively)^15^, ^16^ (Figure 6). An underprediction of the extent of clinical variability was noted for isoniazid, linezolid, and pyrazinamide. The reason for this is not currently clear and additional research specifically into this issue needs to be conducted. There is also a need for more clinical data in order to distinguish the impact of simulation variability in comparison to a need for refinement of the techniques used to obtain the clinical lung concentration data.^20^, ^21^ For rifampicin, use of an optimized value for lung fu_mass_ (increased from 0.058 to 0.116), resulted in a predicted lung‐to‐plasma concentration ratio being in line with that reported by Ziglam et al.^19^ (Figure 2b) and Prideaux et al. ^15^ (Figure S5).

### Simulations of drug distribution to additional lung compartments (epithelial lining fluid, alveolar cells, or tissue) of patients with TB

For 10 of the compounds (clofazimine, cycloserine, ethambutol, ethionamide, isoniazid, kanamycin, linezolid, pyrazinamide, rifampicin, and rifapentine), additional studies that measured drug concentrations in lung tissue alveolar cells and/or epithelial lining fluid (ELF) were identified and simulated data were compared to the observed clinical data (Table S5). Using a preliminary PBPK model, simulated lung tissue mass (ethambutol) or the ELF‐to‐plasma concentration ratio (ethionamide) initially underpredicted observed data.^15^, ^22^ Sensitivity analysis showed that adjusting either lung tissue mass pH or decreasing fu_mass_ (ethambutol) or accounting for P‐glycoprotein activity by scaling available in vitro data^23^ (ethionamide) improved model prediction accuracy. The dibasic nature of ethambutol makes it particularly sensitive to local pH. In contrast for rifapentine, simulated lung tissue mass initially overpredicted observed concentrations (cells collected during alveolar lavage)^24^ using a preliminary PBPK model and sensitivity analysis showed that increasing fu_mass_ (from 0.02 to 0.03) improved simulation of the clinically measured concentrations (Figure 2c).

### Simulations comparing the pharmacokinetics of midazolam and isoniazid in North European Caucasian, South African, and TB patient populations

The impact of demographic and physiological differences among the virtual NEC, Black South African (SA), and Black South African TB (SA‐TB) populations was investigated for midazolam and isoniazid. Midazolam was not modified from the default Simcyp V16 compound file. For the same dose (5 mg), the simulated midazolam AUC_0–24_ was twofold higher in the NEC versus SA population, due to the higher presence of CYP3A5 extensive metabolizers in the SA population (0.82 vs. 0.17). The predicted fraction unbound in plasma (fu_p_) was unchanged between the SA and NEC populations (0.03), however, in the SA‐TB population, the fu_p_ was 0.05, which resulted in a 0.6‐fold lower exposure compared to the general Black South African simulations. Midazolam is highly bound to human serum albumin, which is reduced in patients with TB.^25^

The disposition of isoniazid is altered between individuals based on the NAT‐2 acetylator phenotype. A meta‐analysis of 5382 White individuals shows a proportion of slow, intermediate, and fast acetylator phenotypes of 0.58, 0.37, and 0.05, respectively.^26^ The corresponding phenotype frequencies in the South African population are 0.339, 0.425, and 0.236, respectively (Table S7). Due to the higher proportion of intermediate and fast acetylators in the SA population, isoniazid exposure was lower. The low plasma binding of isoniazid meant that the difference in exposure between the general SA population and patients with TB was negligible, with a slightly higher exposure predicted for patients with TB, mainly driven by reduced renal clearance (major elimination route for isoniazid).

## DISCUSSION

PBPK models have been developed, which are capable of simulating plasma concentrations of 11 standard‐of‐care and newer anti‐TB agents. All PBPK models were able to simulate healthy volunteer plasma *C*_max_ and AUC values to an acceptable degree of accuracy—the simulated mean values were within 1.5‐fold of those reported in the clinical study in the majority of cases. It has been indicated that for comparisons of predicted versus observed exposure of drugs, within twofold of observed data is considered to be “a primary metric for assessment of model fidelity”.^27^

For nine of the compounds (bedaquiline, clofazimine, ethambutol, ethionamide, isoniazid, linezolid, pyrazinamide, rifampicin, and rifapentine), the constructed models provide additional performance verification or the ability to account for additional mechanistic functionality (e.g., to describe DDIs or to simulate lung concentrations).^7^, ^28^, ^29^, ^30^, ^31^, ^32^, ^33^, ^34^ For the other two compounds (cycloserine and kanamycin), the constructed PBPK models are the first to be published in the literature.

The PBPK models for each compound contained a multicompartment permeability‐limited model^7^ of the lungs allowing the concentration of the anti‐TB agents to be simulated in the lung tissue and ELF. For those compounds where lung concentrations have been measured in humans, the simulated concentrations or tissue:plasma ratios were compared with the clinical data.

In addition, elimination and interaction parameters were specified in the PBPK models for rifampicin, rifapentine, and bedaquiline to allow DDIs to be simulated. Observed and simulated DDI ratios were within 1.5‐fold (AUC) and 2‐fold (*C*_max_) of the observed data for nine (AUC) or eight (*C*_max_) of the 10 cases.

The ability of the rifampicin PBPK model to recover the clinically observed magnitude of induction (following co‐administration with a number of sensitive CYP 3A substrates), has been described previously.^35^ Use of the whole body PBPK model with combined permeability‐limited lung model for rifampicin (Figure S1) did not affect the accuracy of predicted extent of interaction for midazolam and triazolam in comparison to use of the default minimal PBPK model for rifampicin (lumping all tissues excluding the intestine, liver, and portal vein; Table 1).

Although PBPK models have been published previously for rifapentine,^28^, ^32^ the current model allows both auto‐induction of arylacetamide deacetylase (AADAC; modeled using a surrogate enzyme) and induction of CYP 3A4 following administration of rifapentine to be simulated. The simulated DDIs between rifapentine and midazolam showed good agreement with the reported magnitude of interaction,^8^ with a slight over prediction of the change in *C*_max_. In addition, the interaction between multiple doses of rifampicin and bedaquiline were simulated with an acceptable degree of accuracy, showing the potential of using PBPK for simulating different dosage regimens of these drugs in the future. The DDI liability of bedaquiline when administered with ketoconazole (a strong CYP 3A inhibitor) was also adequately simulated using the PBPK model. In contrast, the changes in exposure to the *N*‐desmethyl metabolite of bedaquiline were simulated with less precision. The contribution of CYP 3A4 to the elimination of *N*‐desmethyl bedaquiline is not known and incorporation of this information may allow further improvement of the PBPK model for this important active metabolite of bedaquiline. The ability to account for potential interactions between co‐administered drugs is important in designing effective therapeutic TB regimes as currently, treatment involves administration of multiple doses of a number of drugs concomitantly.

An important question is what should be considered a successful prediction for drug tissue concentrations? In the initial development of the mechanistic tissue composition equations proposed by Rodgers and co‐workers, a prediction of tissue:plasma ratio was considered acceptable if it was predicted within a factor of 3.^12^, ^13^, ^14^ In a more recent study, which examined the ability of in silico models to predict drug distribution into human tissues, a prediction was considered successful if it was within 2.5‐fold of the observed values.^36^ An additional complication with the clinical data used for comparative purposes in this study, is that there was a high degree of interstudy, intersubject, and within‐subject (from different samples in the same individual) variability. This variability partly arises from the actual procedure to measure the lung concentration of anti‐TB drugs, which is technically challenging and not routine. The caveats and challenges that arise when using measured concentrations of drugs in ELF, as a measure of lung penetration of compounds, have been eloquently discussed by Kiem and co‐workers.^20^, ^21^ In this study, predictions were considered successful if the predicted mean data were within 2.5‐fold of the observed data. For individual timepoint data, predictions were considered successful if the majority of the observed data were within the simulated 5th–95th percentiles. Based on these criteria, lung concentrations could be predicted with an acceptable degree of accuracy for six of the nine compounds where clinical data were available for comparison.

For a number of compounds, the final models represent the culmination of model refinement steps (Figure 5). In particular, the initial PBPK models (prior to refinement) poorly predicted the measured lung concentrations for cycloserine, ethionamide, and rifapentine. Possible reasons for these initial mispredictions of tissue concentrations are discussed below.

The permeability of cycloserine into the lung tissue was initially overpredicted, leading to an overprediction of lung concentrations. Predicted fu_mass_ was ~ 1 so it could not be contributing to this prediction inaccuracy. The physicochemical properties of cycloserine are outside the range of the compounds used to construct the quantitative structure activity relationship (QSAR) model to predict permeability. Obtaining experimental permeability values for this compound may improve the prediction accuracy. In addition, four out of six measured cycloserine lung tissue mass concentrations were below the stated LLOQ of the assay^17^ and data were only available from this one clinical study for this compound. For ethionamide, it was necessary to include the action of the P‐glycoprotein transporter (moving drug from the lung tissue mass to ELF), in order to correctly predict the ELF concentration.^37^, ^38^

Due to a lack of observed lung concentration data for rifapentine or linezolid, PBPK model accuracy was assessed using observed plasma and ELF concentrations. For rifapentine, plasma concentrations over time were reasonably simulated under different dosing scenarios. However, the simulated ELF:plasma ratio underpredicted the data from the single clinical study (where this parameter has been measured^24^) by a factor of sixfold. This is perhaps not surprising given that reported ELF:plasma ratio for rifapentine is similar to that reported for the related compound rifampicin, despite the plasma unbound fraction (fu) of rifapentine being 10‐fold lower. Sensitivity analysis showed that the PBPK model was sensitive to some of the uncertain parameters in the model including the fu_ELF_, the ELF pH and the physicochemical properties of rifapentine. Optimization of these parameters resulted in simulated ELF: plasma ratios being within the range of the reported clinical data. Further experimental work is needed to build confidence in these values. As Kiem et al. have discussed, the bronchoalveolar lavage technique to ascertain ELF concentrations can be subject to experimental error^20^, ^21^ and there is also a lack of reliable clinical data sources available.

The ELF:plasma ratios of linezolid were simulated and compared with clinical data obtained in four different studies^39^, ^40^, ^41^, ^42^ (Figure S4). Simulation ratios were comparable to those reported by Boselli et al.^39^, ^40^ but underpredicted those measured by Honeybourne et al.,^42^ and Conte et al.^41^ It is not clear whether this clinical variability is due to differences in methodology (i.e., mini BAL Boselli et al. vs. BAL Conte et al. and Honeybourne et al. measurements) or patient population differences.

Although local sensitivity analyses were conducted for each compound, a common theme for all compounds was that simulated total drug lung tissue concentration was sensitive to the binding of drug in the lung tissue. This is not surprising, because the compounds studied (apart from cycloserine and kanamycin) were predicted to have high permeability between the blood and lung tissue. The predicted concentrations for the majority of compounds were not sensitive to changes in permeability over a factor of 100 (10‐fold lower or higher than predicted).

Use of either a predicted (0.00019) or optimized (0.01) value for clofazimine fu_mass_, allowed recovery of clinical data reported by either Prideaux et al.^15^ or Dheda et al.,^17^ respectively. However, as the measured lung tissue concentrations differed by a factor of 1000, it is not feasible to simulate both sets of data with a single PBPK model. As all observed clofazimine data points were below the LLOQ for the assay described by Dheda et al.,^17^ simulated clofazimine lung concentrations are presented that correspond to use of the value predicted (0.00019), using the mechanistic tissue composition approach described by Rodgers, Rowland, and co‐workers.^12^, ^13^, ^14^ It should be considered that these equations are sensitive to the lipophilicity of the compound and are therefore, less accurate for highly lipophilic compounds such as clofazimine (log P ~7.4).

An important consideration for all PBPK models is that, although it is possible to change the total concentration within the lung tissue compartment by adjusting the binding in the lung (fu_mass_), these changes do not have a pronounced effect on the unbound concentration within the lungs, which is the concentration generally assumed to drive PD efficacy or toxicity.

An additional factor that needs to be considered, is that physiological, demographic, and biochemical data used for the PBPK model, should reflect the characteristics of the target population. In this study, observed data from patients mainly located in South Korea were compared with PBPK model outputs using the Sim‐North European Caucasian virtual population.^15^ This is a limitation of the study and future work will involve assessment of data availability to develop a PBPK population specific to South Korea.

A first step toward developing a PBPK population for South African patients with TB is detailed in the Supplementary Material. Due to data limitations, it was not possible to identify all physiology data in studies that only considered South African patients with TB (as described in the Supplementary Material). Therefore, the default NEC Simcyp library values were used to provide estimates for some of the physiological parameters (e.g., liver volume, kidney density, concentrations of human serum albumin, and blood α‐acid‐glycoprotein). The TB population was used to compare simulations with clinical data provided by Dheda et al.^17^ For both midazolam (CYP 3A4/5 substrate) and isoniazid (NAT‐2 substrate), PK differences were simulated for the SA‐TB population in comparison to the European Caucasian population (Figure 6). In patients with TB, changes in a number of parameters, including body weight, plasma proteins, and hematocrit, were noted when compared with healthy individuals. As the fraction unbound in blood is influenced by both plasma protein concentration and hematocrit, measured values for these parameters in the clinical population would be beneficial. Further studies are needed to refine the developed TB population and to verify the simulated PKs for a range of drugs and comedication regimes.

## CONFLICT OF INTEREST

H.H., L.A., I.G., O.H., X.P., B.S., M.J., and M.Z. are employees of Certara UK Limited (Simcyp Division). A.B. and K.R. are past and current employees of the Critical Path Institute.

## AUTHOR CONTRIBUTIONS

H.H., A.B., K.R., I.G., O.H., M.Z., L.A., B.S., and X.P. wrote the manuscript. I.G., K.R., A.B., H.H., and M.J. designed the research. H.H., I.G., M.Z., X.P., and O.H. performed the research. H.H., I.G., M.Z., X.P., and O.H. analyzed the data.

## Supporting information

Supplementary Material

Figures S1–S5

Table S1–S5
