# Supplementary material for: Development of physiologically‐based pharmacokinetic models for standard of care and newer tuberculosis drugs
Source: CPT Pharmacometrics Syst Pharmacol. 2021 Oct 8;10(11):1382–95. doi: 10.1002/psp4.12707 (PMC8592506; doi:10.1002/psp4.12707)
Supplement: Supplementary file 1 — Supplementary Material [file PSP4-10-1382-s003.docx]

**Development of a Black South African and Black South African TB population**

In order to investigate the disposition of TB drugs in studies utilising an adult South African population, a virtual South African population was developed. Data for age-population distributions (by gender) reported in the South-African 2011 Census Survey conducted by Statistics South Africa ^1^, were fitted to a Weibull age distribution for individual’s ≥ 20 years of age (Figure 1). The age distribution of TB infected patients was fitted to a Weibull distribution based on the South African General Household Survey, 2011 ^2^ (Figure 1).

Data describing body height and age in the adult South-African population were taken from the South-African Demographics and Health Survey in 2003 ^3^. The relationship between age and height was investigated and found to be described by a quadratic equation for both males and females (Equations 1&2, Figure 2). A CV of 7.33 and 5.83% was applied to capture variability in male and female data, respectively.

Male: Height(cm) = 1.62 X 10^2^ + 3.59 X 10^-1^ * Age (y) –4.29 X 10^-3^ * (Age(y)^2^) [Eq. 1]

Female: Height(cm) = 1.55 X 10^2^ + 2.07 X 10^-1^ * Age (y) -2.68 X 10^-3^ * (Age(y)^2^) [Eq. 2]

Data describing body height and weight in healthy adult South-African ^3, 4^ populations were obtained. These data did not have individual data points and so were reserved for model verification.

To build the model, individual height and weight data obtained from Dr Collen Masimirembwa was used. These data were collected from individuals of the Shona tribe in Zimbabwe and included individuals from urban and rural communities. In total there were 124 female and 60 male subjects from an urban environment who were HIV infected, 59 female and 27 male subjects from a rural area who were HIV infected and 30 healthy males from an urban environment. In total, this gave 183 females and 117 male subjects. Other studies did not show significant differences in weight between individuals with and without HIV infection ^4^ and so all of the data were pooled. The relationship for male and females is described by equation 3 and 4, with a CV of 21.1 and 26.4%, respectively. Observed vs simulated weight-height relationships for male and females are shown in Figure 3.

Male: Ln weight = 2.97 + 0.007*height [Eq.3]

Female: Ln weight = 3.19 + 0.007*height [Eq.4]

The impact of TB infection indicates a reduction of body weight in comparison to healthy individuals. Updated weight-height relationships were utilised (Equations 5 and 6). Using these values, the average weight in females was 61 kg (86% of the value in the healthy population) and 59.4 kg in males (87% of the value in the healthy population). In addition, the mean BMI was 24.1 kg/m^2^ in females and 21 kg/m^2^ in males. These values are consistent with values reported in the literature for TB infected individuals ^5, 6, 7, 8, 9, 10^. Identical CV values were assumed as healthy black South African data.

Male: Ln weight = 2.79 + 0.0073*height [Eq.5]

Female: Ln weight = 2.998 + 0.0067*height [Eq.6]

Estimates of body surface area in African subjects have been shown to be under-determined by ~ 6 – 22 % when using the DuBois & DuBois equation or modifications of this formula ^11^. Instead an equation (Equation 7) based on male, 18 – 55 year-old, non-obese, Nigerian population (n=20) was assessed to predict South African data. The performance is shown in Figure 4.

BSA (m^2^) = Weight^(0.2620)^ x 0.001315 x Height^(1.2139)^ [Eq.7]

Organ volumes were assessed with available literature data. Data for Liver volumes were inadequate for quantitative assessment, and therefore North European Caucasian was assumed as a default. The kidney weight in individuals from different tribes in South Africa has been reported ^12^. On this basis, Kidney weight relationship was devised (Equation 8). Based on the observed data the CV was 4%. Kidney density was assumed based on North European Caucasian data.

Kidney weight = 15.4 + 1.7*Weight x 36*Height

Simulations in the Simcyp population (1000 individuals) gave an average of 0.98 million nephrons (range 0.76 – 1.41), a reasonable estimate compared to observed 0.94 million nephrons (range 0.54-1.76) in a Senegalese population ^13^.

All other organ volumes were assumed to be calculated based on relationships established in North European Caucasians.

Human serum albumin (HSA) abundances were reasonably simulated for urban South African populations using the default parameters from the North Caucasian European population Watermeyer et al., ^14^. In the absence of literature data, concentration of blood α-acid-glycoprotein was assumed to be the same as North European Caucasian.

The concentration of albumin and total serum protein are known to decrease in individuals infected with TB. Morris measured serum albumin concentration in 265 black South African individuals with TB and the mean value (+/- SD) was 31+/-6.9 g/L ^15^. This value is about 0.7 of the average value in a healthy population. A meta-analysis of HSA concentration in TB infected individuals (n=1212) gave values of 32 +/- 7 g/L (mean +- SD).

No studies were found describing blood concentration of α-acid-glycoprotein (AGP) in TB infected South African individuals. However, a number of studies were identified where AGP concentration had been measured in subjects infected with TB. The average concentration of AGP in TB infected individuals was 2.3-fold higher than in healthy individuals. AGP levels in TB infected SA patients were therefore 1.865 and 1.819 g/L for male and female, respectively, with an assumed CV of 30% used for both male and female populations.

Literature searches identified three studies where the haemoglobin concentrations in subjects with TB were identified. In total these papers had data for 346 individuals. The largest study was by Morris et al., ^15^ and looked at TB in Black South African individuals. The mean haemoglobin concentration was 11.5 (CV 20%) g/dl in male subjects with TB and 11.0 (CV 20%) g/dl in females with TB. In the same paper the quoted normal range for haemoglobin concentration in Black South Africans is 13 -16 g/dl in males and 11-14 g/dl in females. Converting to haematocrit give ranges of 39-48% in healthy male African subjects and 33-42% in healthy female individuals. Based on these values the haematocrit in healthy Black South African females (Figure) and males (Figure) is adequately described by the existing algorithms within Simcyp.

Data for cardiac output from Nigerian individuals were used as a surrogate for South African individuals ^16, 17, 18^. Using the default algorithms, cardiac output was under predicted, and therefore a cardiac output scalar of 1.04 was used.

**Drug Metabolising Enzyme Phenotype Frequency**

A number of drug metabolising enzymes including CYP 3A5, 2B6, 2C9, 2C19, 2D6 and NAT-2 are known to be polymorphically expressed in the South African population. Inter-individual variability in the activity of polymorphic enzymes could influence the pharmacokinetics and drug-drug interactions of drugs targeted at TB infection. The pharmacogenetics of CYP 3A5 is more complex in African populations than it is in the Caucasian population. In addition to a poor metaboliser phenotype arising from individuals having two *3 alleles in African populations additional poor metaboliser genotypes exist (*3/*6, *3/*7, *6/*6, *6/*7) that are rarely expressed in the North European Caucasian population. An additional complexity is that not all of these genotypes have been looked for in all of the published studies looking at CYP 3A5 genotypes in black South African individuals. To overcome these limitations a meta-analysis was conducted independently for each of the poor metaboliser genotypes and the frequency of the different combinations summed to give the percentage of poor metabolisers in the population. The frequency of the poor metaboliser phenotype in Black South African subjects was 18.2% ^19, 20, 21^. The Black South African population has a much higher frequency of CYP 3A5 extensive metaboliser individuals (0.82) compared to Caucasian populations (0.17). EM and PM frequencies for other CYP enzymes are summarised in Table S4.

**Table S6. Summary of CYP phenotype frequencies**

| **Study** | **n** | **Ethnicity** | **Phenotype/Genotype** | **EM Frequency** | **PM Frequency** |
| --- | --- | --- | --- | --- | --- |
| **CYP2B6** |  |  |  |  |  |
| Gounden *et al* (2010) ^22^ | 80 | Black South African | Phenotype | 0.775 | 0.225 |
| Matimba *et al* (2008) ^23^ | 153 | Tanzanian | Phenotype | 0.848 | 0.152 |
|  | 100 | Shona (Zimbabwe) | Phenotype | 0.856 | 0.144 |
|  | 81 | Venda (South Africa) | Phenotype | 0.87 | 0.13 |
| Viljoen *et al* (2012) ^24^ | 60 | Black South African | Phenotype | 0.77 | 0.23 |
| Ngaimisi *et al* (2013) ^25^ | 183 | Tanzanians | Phenotype | 0.814 | 0.186 |
| Haas et al (2009) ^26^ | 34 | African-American | Phenotype | 0.794 | 0.206 |
| Swart *et al* (2013) ^27^ | 163 | South African | Phenotype | 0.896 | 0.104 |
| SUM | 854 |  |  |  |  |
| Weighted mean | 0.835 | 0.144 |  |  |  |
| **CYP2C9** |  |  |  |  |  |
| Mitchell *et al* (2011) ^28^ | 100 | South African (ethnicity not known) | Phenotype (Alleles: *5, *9, *6, *32 ) |  | 0.026 |
| Man *et al* (2010) ^29^ | 250 | ‘African’# | Phenotype (Alleles: *6, *11, *8, *9 ) |  | 0.009 |
| Matimba *et al* (2009) ^30^ | 93 | African ethnic groups## | Phenotype (Alleles: *5, *6, *9, *31, *32 ) |  | 0.035 |
| Sistonen *et al* (2009) ^31^ | 204 | African (excluding Northern Africa)### | Phenotype (Alleles: *2, *3, *5, *11 ) |  | 0.056 |
| SUM | 647 |  |  |  |  |
| Weighted mean |  |  |  |  | 0.024 |
| **CYP2C19** |  |  |  |  |  |
| Man et al (2010) ^29^ | 250 | ? African-American | Phenotype | 0.956 | 0.044 |
| Martis *et al* (2013) ^32^ | 250 | African American | Phenotype | 0.948 | 0.052 |
| Dandara *et al* (2001) ^33^ | 106 | Tanzanian | Phenotype | 0.962 | 0.038 |
|  | 76 | Venda | Phenotype | 0.947 | 0.053 |
|  | 84 | Zimbabwean | Phenotype | 0.965 | 0.035 |
| Dandara *et al* (2011) ^20^ | 993 | Black South African | Phenotype | 0.97 | 0.03 |
| Drogemoller *et al* (2010) ^34^ | 100 | Xhosa (South African) | Phenotype | 0.97 | 0.03 |
| Drogemoller *et al* (2010) ^34^ | 75 | Colored (South African) | Phenotype | 0.92 | 0.08 |
| SUM | 1934 |  |  |  |  |
| Weighted mean |  |  |  | 0.9618 | 0.0382 |
| **CYP2D6** |  |  |  |  |  |
| Dandara *et al*., (2001) ^33^ | 76 | South African (Venda) | Genotype |  | 0.026 |
|  | 114 | Zimbabwean | Genotype |  | 0.009 |
| Masimirembwa *et al*., (1996) ^35^ | 103 | Zimbabwean | Phenotype *(DSQ 0.02, MET 0.05)* |  | 0.035 |
| Sommers et al., (1989) ^36^ | 98 | South African (Venda) | Phenotype *(DSQ 0.04, MET 0.075 n=94)* |  | 0.056 |
| SUM | 391 |  |  |  |  |
| Weighted mean |  |  |  |  | 0.031 |

#Ambiguous ethnicity. Study carried out in America, therefore, African-American is perhaps most likely

##Ethnic groups include Hausa, Luo, Maasa, San, Shona, Venda, Bantu

### Only 15 individuals specifically from South Africa were included in the study which was too few to detect any variants. An average of all Africa (excluding Northern Africa) was deemed a reasonable alternative. Northern African was excluded owing to the Arabic influence of Northern African countries.

N-acetyltransferase (NAT-2) is involved in the metabolism of isoniazid, and can be classified into fast, intermediate or slow acetylator type. In a comparison of genotypes between a Caucasian and Black South African population it was noted that diversity of alleles is much greater in the South African population. Table S5 summarises the NAT-2 phenotypic frequencies. The relative activity of NAT-2 in fast, intermediate and slow acetylators was calculated to be 1.0, 0.61 and 0.29, based on isoniazid oral clearance observed in NAT-2 phenotyped SA patients with pulmonary TB (Donald et al 2007).

**Table S7 Phenotype Frequencies for N-acetyltransferase (NAT-2) in Black South African population**

| **Ref.** | **n** | **Genotype/ Phenotype** | **Country** | **Subjects/ Ethnicity** | **% Phenotype** |  |  |
| --- | --- | --- | --- | --- | --- | --- | --- |
|  |  |  |  |  | **Slow** | **Inter** | **Fast** |
| Donald et al., 2004 ^37^ | 87 | Gene | South Africa (Stellenbosch) | TB+, West cape | 32.2 | 41.4 | 26.4 |
| Zhu et al., 2012 ^38^ | 151 | Gene | South Africa (Stellenbosch) | Infants Cape Town/ Durban | 31.8 | 44.4 | 23.8 |
| Parkin et al., 1997 ^39^ | 47 | Gene | South Africa (Stellenbosch) | Coloured or mixed race | 31.9 | 44.7 | 23.4 |
| Loktionov et al., 2002 ^40^(44) | 101 | Gene | South Africa (Potchefstroom) | Tswana speaking, Black | 39.6 | 39.6 | 20.8 |
| **Sum** | **386** |  |  | **Weighted Mean** | **33.9** | **42.5** | **23.6** |

References

1. Statistics South Africa. Census 2011 Census in brief Report No. 03-01-41. 2012.

2. Statistics South Africa. Use of health facilities and levels of selected health conditions in South Africa: Findings from the General Household Survey, 2011 Report No. 03–00–05. 2013.

3. South African Medical Research Council. South Africa Demographic and Health Survey 2003. 2003 18 Feburary 2015 [cited 2015 13 March 2015]Available from: http://www.mrc.ac.za/bod/sadhs.htm

4. Malaza A, Mossong J, Bärnighausen T, Newell M-L. Hypertension and obesity in adults living in a high HIV prevalence rural area in South Africa. *PLoS ONE* **7** e47761-e47761. (2012)

5. Sanchez A, Azen C, Jones B, Louie S, Sattler F. Relationship of Acute Phase Reactants and Fat Accumulation during Treatment for Tuberculosis. *Tuberc Res Treat* **2011** 346295. (2011)

6. Long R*, et al.* Pulmonary tuberculosis treated with directly observed therapy: serial changes in lung structure and function. *Chest* **113** 933-943. (1998)

7. Karyadi E*, et al.* Poor micronutrient status of active pulmonary tuberculosis patients in Indonesia. *J Nutr* **130** 2953-2958. (2000)

8. Matos ED, Moreira Lemos AC. Association between serum albumin levels and in-hospital deaths due to tuberculosis. *Int J Tuberc Lung Dis* **10** 1360-1366. (2006)

9. Kim DK*, et al.* Nutritional deficit as a negative prognostic factor in patients with miliary tuberculosis. *Eur Respir J* **32** 1031-1036. (2008)

10. Kim HJ*, et al.* The impact of nutritional deficit on mortality of in-patients with pulmonary tuberculosis. *Int J Tuberc Lung Dis* **14** 79-85. (2010)

11. Nwoye LO. Body surface area of Africans: a study based on direct measurements of Nigerian males. *Hum Biol* **61** 439-457. (1989)

12. Moar JJ, Reinach SG. Renal weights in the Southern African black population. *American Journal of Physical Anthropology* **76** 105-110. (1988)

13. McNamara BJ*, et al.* A comparison of nephron number, glomerular volume and kidney weight in Senegalese Africans and African Americans. *Nephrol Dial Transplant* **25** 1514-1520. (2010)

14. Watermeyer GS, Solomon L, Daynes G, Soskolne CL, Beighton PH. The changing epidemiology of serum albumin levels in Southern Africa. *S Afr Med J* **51** 614-616. (1977)

15. Morris CD, Bird AR, Nell H. The haematological and biochemical changes in severe pulmonary tuberculosis. *Q J Med* **73** 1151-1159. (1989)

16. Akintunde AA, Familoni OB, Akinwusi PO, Opadijo OG. Relationship between left ventricular geometric pattern and systolic and diastolic function in treated Nigerian hypertensives. *Cardiovasc J Afr* **21** 21-25. (2010)

17. Lasisi GT, Adebola AP, Ogah OS, Daniel FA. Prevalence of ventricular arrhythmias and heart rate variability pattern in chronic heart failure. *Niger Postgrad Med J* **19** 157-162. (2012)

18. Schutte AE*, et al.* Inflammation, obesity and cardiovascular function in African and Caucasian women from South Africa: the POWIRS study. *J Hum Hypertens* **20** 850-859. (2006)

19. Dandara C, Ballo R, Parker MI. CYP3A5 genotypes and risk of oesophageal cancer in two South African populations. *Cancer Lett* **225** 275-282. (2005)

20. Dandara C*, et al.* Genetic variants in CYP (-1A2, -2C9, -2C19, -3A4 and -3A5), VKORC1 and ABCB1 genes in a black South African population: a window into diversity. *Pharmacogenomics* **12** 1663-1670. (2011)

21. Fernandez P*, et al.* Androgen Metabolism Gene Polymorphisms, Associations with Prostate Cancer Risk and Pathological Characteristics: A Comparative Analysis between South African and Senegalese Men. *Prostate Cancer* **2012** 798634. (2012)

22. Gounden V, van Niekerk C, Snyman T, George JA. Presence of the CYP2B6 516G> T polymorphism, increased plasma Efavirenz concentrations and early neuropsychiatric side effects in South African HIV-infected patients. *AIDS Res Ther* **7** 32. (2010)

23. Matimba A*, et al.* Establishment of a biobank and pharmacogenetics database of African populations. *Eur J Hum Genet* **16** 780-783. (2008)

24. Viljoen M*, et al.* Influence of CYP2B6 516G>T polymorphism and interoccasion variability (IOV) on the population pharmacokinetics of efavirenz in HIV-infected South African children. *Eur J Clin Pharmacol* **68** 339-347. (2012)

25. Ngaimisi E*, et al.* Importance of ethnicity, CYP2B6 and ABCB1 genotype for efavirenz pharmacokinetics and treatment outcomes: a parallel-group prospective cohort study in two sub-Saharan Africa populations. *PLoS ONE* **8** e67946. (2013)

26. Haas DW*, et al.* Associations between CYP2B6 polymorphisms and pharmacokinetics after a single dose of nevirapine or efavirenz in African americans. *J Infect Dis* **199** 872-880. (2009)

27. Swart M*, et al.* High predictive value of CYP2B6 SNPs for steady-state plasma efavirenz levels in South African HIV/AIDS patients. *Pharmacogenet Genomics* **23** 415-427. (2013)

28. Mitchell C, Gregersen N, Krause A. Novel CYP2C9 and VKORC1 gene variants associated with warfarin dosage variability in the South African black population. *Pharmacogenomics* **12** 953-963. (2011)

29. Man M*, et al.* Genetic variation in metabolizing enzyme and transporter genes: comprehensive assessment in 3 major East Asian subpopulations with comparison to Caucasians and Africans. *J Clin Pharmacol* **50** 929-940. (2010)

30. Matimba A, Del-Favero J, Van Broeckhoven C, Masimirembwa C. Novel variants of major drug-metabolising enzyme genes in diverse African populations and their predicted functional effects. *Hum Genomics* **3** 169-190. (2009)

31. Sistonen J*, et al.* Pharmacogenetic variation at CYP2C9, CYP2C19, and CYP2D6 at global and microgeographic scales. *Pharmacogenet Genomics* **19** 170-179. (2009)

32. Martis S*, et al.* Multi-ethnic distribution of clinically relevant CYP2C genotypes and haplotypes. *Pharmacogenomics J* **13** 369-377. (2013)

33. Dandara C*, et al.* Genetic polymorphism of CYP2D6 and CYP2C19 in east- and southern African populations including psychiatric patients. *Eur J Clin Pharmacol* **57** 11-17. (2001)

34. Drogemoller BI*, et al.* Characterization of the genetic profile of CYP2C19 in two South African populations. *Pharmacogenomics* **11** 1095-1103. (2010)

35. Masimirembwa C*, et al.* Phenotype and genotype analysis of debrisoquine hydroxylase (CYP2D6) in a black Zimbabwean population. Reduced enzyme activity and evaluation of metabolic correlation of CYP2D6 probe drugs. *Eur J Clin Pharmacol* **51** 117-122. (1996)

36. Sommers DK, Moncrieff J, Avenant J. Non-correlation between debrisoquine and metoprolol polymorphisms in the Venda. *Hum Toxicol* **8** 365-368. (1989)

37. Donald PR*, et al.* The influence of human N-acetyltransferase genotype on the early bactericidal activity of isoniazid. *Clin Infect Dis* **39** 1425-1430. (2004)

38. Zhu R*, et al.* The pharmacogenetics of NAT2 enzyme maturation in perinatally HIV exposed infants receiving isoniazid. *J Clin Pharmacol* **52** 511-519. (2012)

39. Parkin DP*, et al.* Trimodality of isoniazid elimination: phenotype and genotype in patients with tuberculosis. *Am J Respir Crit Care Med* **155** 1717-1722. (1997)

40. Loktionov A*, et al.* Differences in N-acetylation genotypes between Caucasians and Black South Africans: implications for cancer prevention. *Cancer Detect Prev* **26** 15-22. (2002)
