# Supplementary material for: Development of physiologically‐based pharmacokinetic models for standard of care and newer tuberculosis drugs
Source: CPT Pharmacometrics Syst Pharmacol. 2021 Oct 8;10(11):1382–95. doi: 10.1002/psp4.12707 (PMC8592506; doi:10.1002/psp4.12707)
Supplement: Supplementary file 2 — Figures S1–S5 [file PSP4-10-1382-s001.pdf]

Figure S1

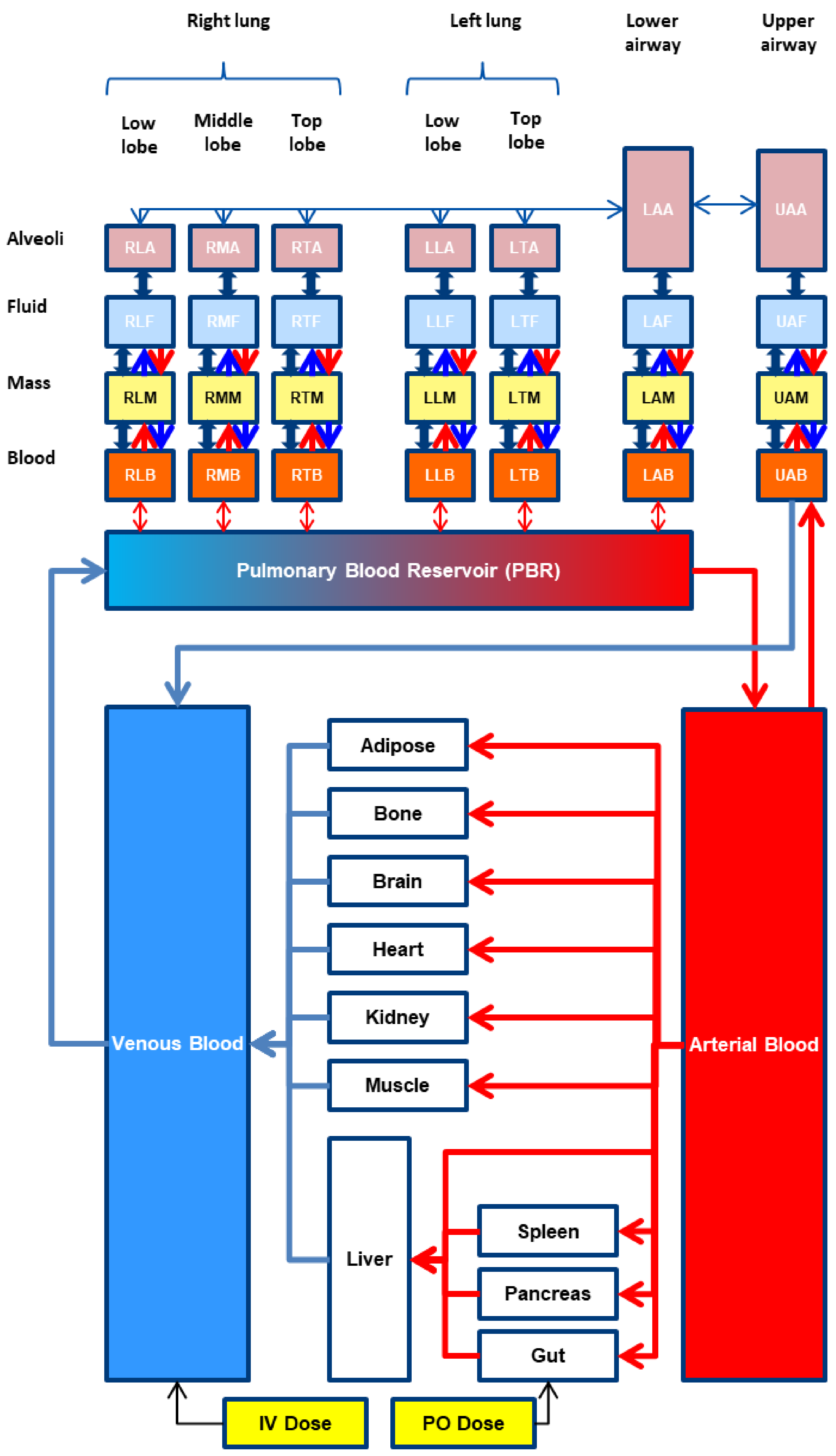

Figure S1. Structure of the multicompartment permeability-limited lung model embedded in the Simcyp full-PBPK model, which consists of 12 perfusion-limited tissue compartments, in addition to the lung. Figure taken from previously published manuscript (L Gaohua, J Wedagedera, BG Small, L Almond, K Romero, D Hermann, D Hanna, M Jamei, I Gardner, 2015. Development of a Multicompartment Permeability-Limited Lung PBPK Model and Its Application in Predicting Pulmonary Pharmacokinetics of Antituberculosis Drugs. CPT Pharmacometrics Syst. Pharmacol. 4: 605-613. https://ascpt.onlinelibrary.wiley.com/doi/abs/10.1002/psp4.12034. https://creativecommons.org/licenses/by-nc-nd/4.0/#). The lung is approximated by seven segments, namely, upper (UA) and lower (LA) airways, left lung top lobe (LT), left lung lower lobe (LL), right lung top lobe (RT), right lung middle lobe (RM), and right lung low lobe (RL). Each of the segments contains four compartments representing pulmonary capillary blood (B), pulmonary tissue mass (M), fluid (F), and alveoli (A). Equilibrium is assumed between the fluid and the alveolar air. Within the lung model, the double arrows represent bidirectional passive permeability between adjacent compartments within the same segment, whereas the unidirectional arrows represent the active transport across the basal and apical membranes of pulmonary tissue. Metabolic elimination exists in the mass compartments.

Figure S2

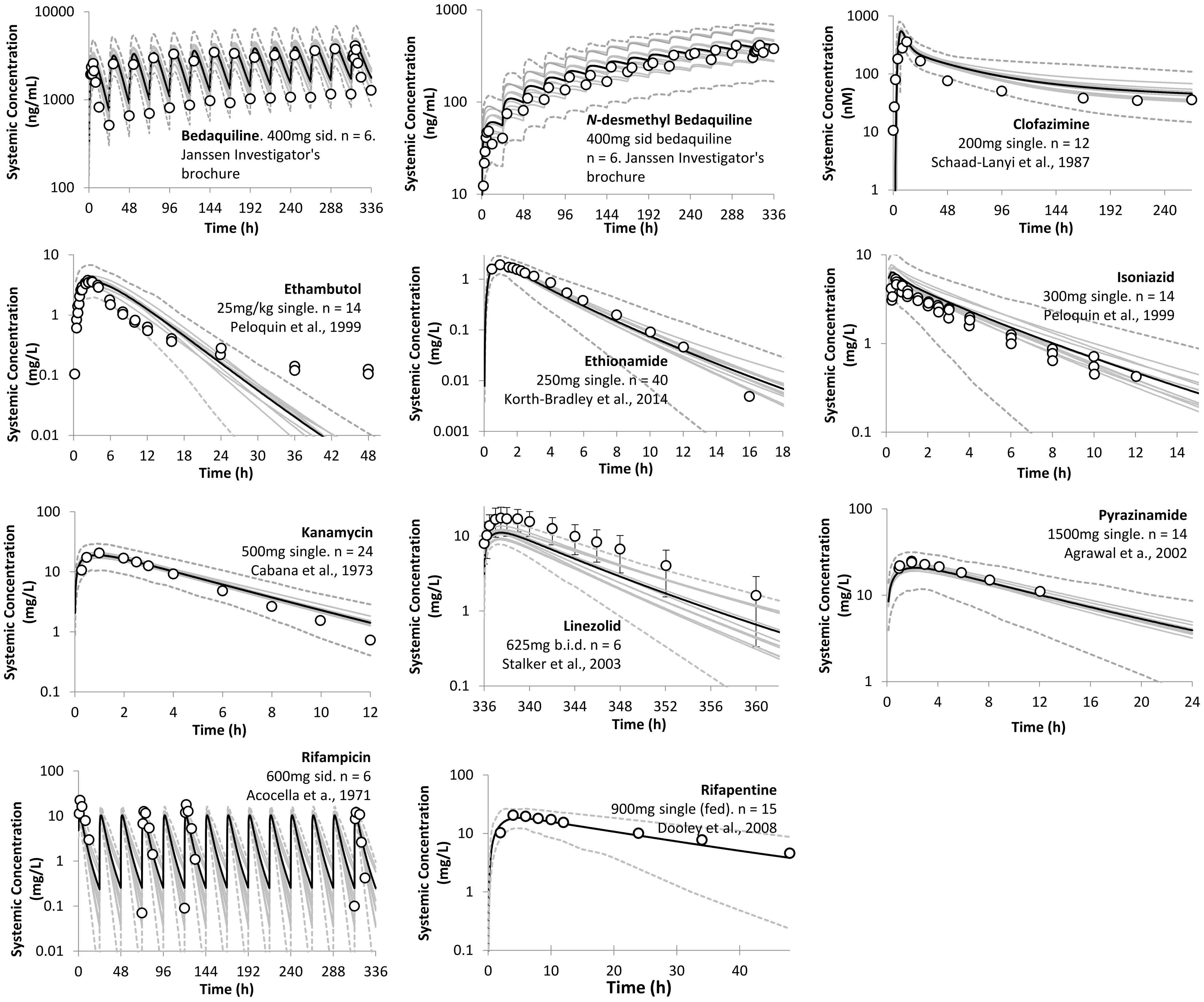

Figure S2. Simulated versus observed concentration in plasma over time for 10 drugs administered to healthy volunteers. Simulated (lines) and observed (data points) plasma concentration over time for bedaquiline, N-desmethyl bedaquiline, clofazimine, ethambutol, ethionamide, isoniazid, kanamycin, linezolid, pyrazinamide, rifampicin and rifapentine. The short dashed lines represent the 5th and 95th percentile of the prediction in the total virtual population (10 trials of n number of healthy volunteers. Age range and proportion of females matched the clinical study)

Figure S3

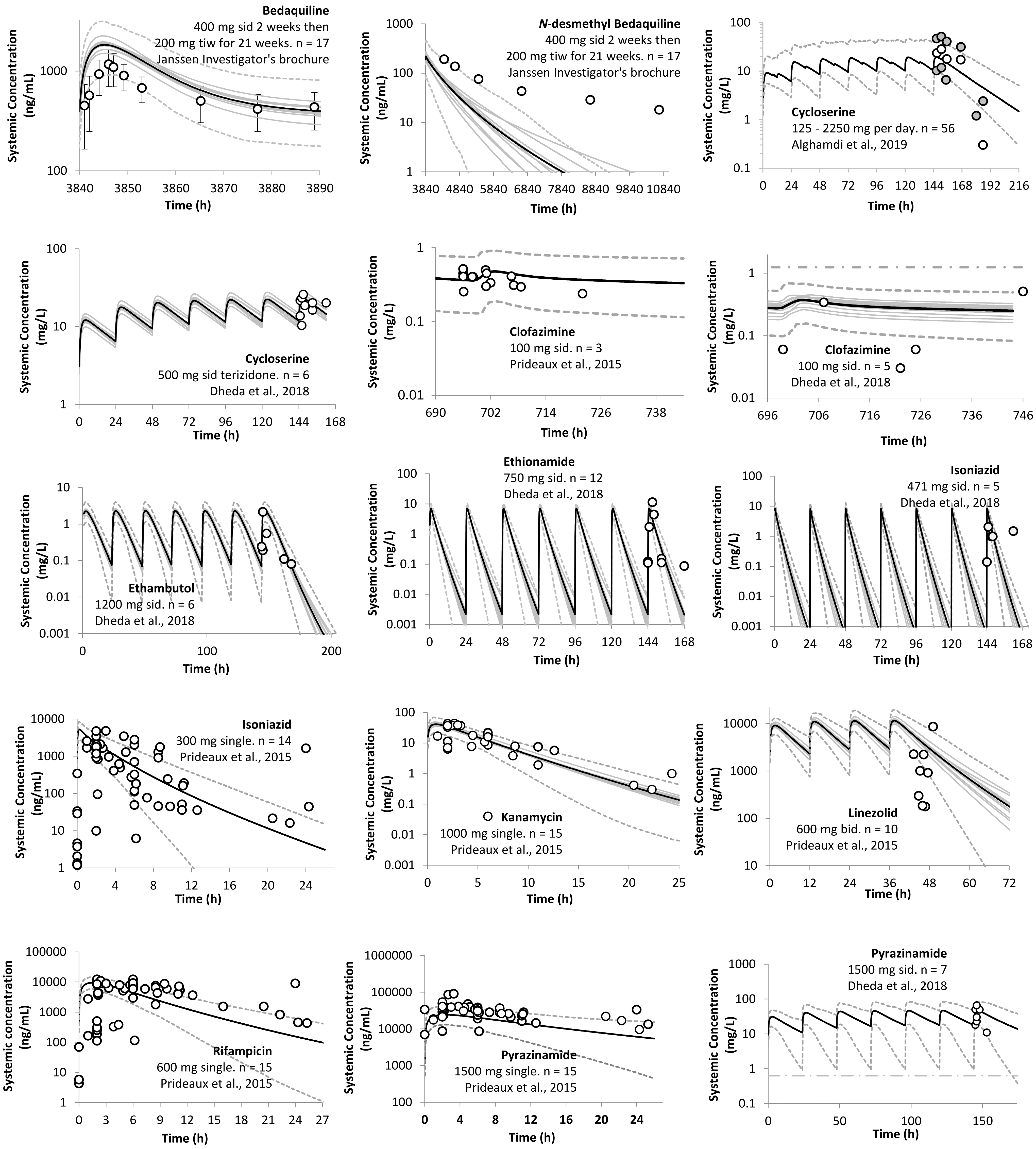

Figure S3. Simulated versus observed concentration in plasma over time for 10 drugs administered to TB patients. Simulated (lines) and observed (data points) plasma concentration over time for bedaquiline, N-desmethyl bedaquiline, cycloserine, clofazimine, ethambutol, ethionamide, isoniazid, kanamycin, linezolid, rifampicin and pyrazinamide. The short dashed lines represent the 5th and 95th percentile of the prediction in the total virtual population (10 trials of n number of healthy volunteers. Age range and proportion of females matched the clinical study). The long dash/dotted lines represent the LLOQ for Dheda et al., 2018.

Figure S4

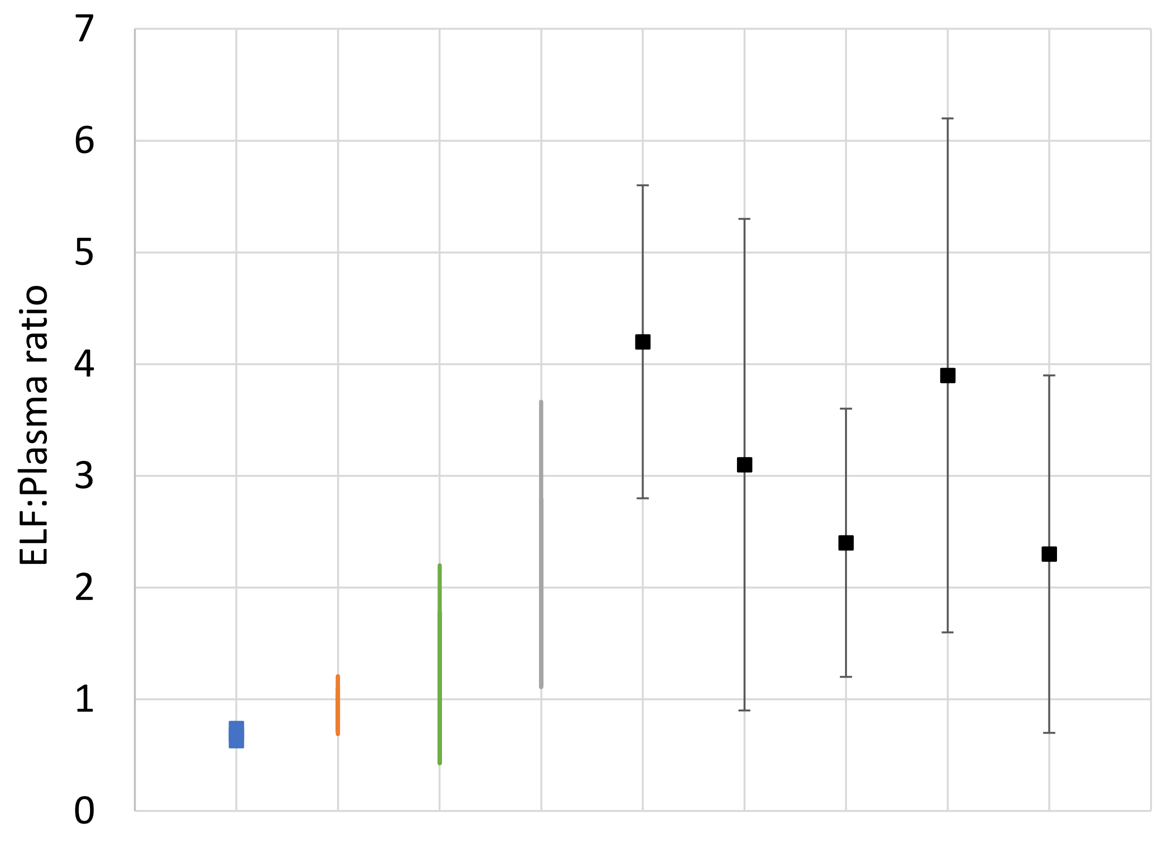

Figure S4. Simulated (blue bar) and observed (orange, green, grey and black squares) Linezolid ELF: plasma ratios. The simulated ELF: plasma ratios are presented as the range of simulated values. The orange line is the range of ELF: plasma ratios reported by Boselli et al 2012, the green line is the range of values reported by Boselli et al 2005 (only the data at 24 hours is used as the Cmax in plasma was at a different time to the ELF sampling and therefore a true comparison for this data cannot be made), the grey line is the range of values reported by Honeybourne et al and the data from Conte et al is reported as the mean +/SD with data at each of the measured timepoints shown. The timepoints in the study by Conte etal., 2003 are from left to right 4, 8, 12, 24 and 48 hours after the last dose of Linezolid.

Figure S5

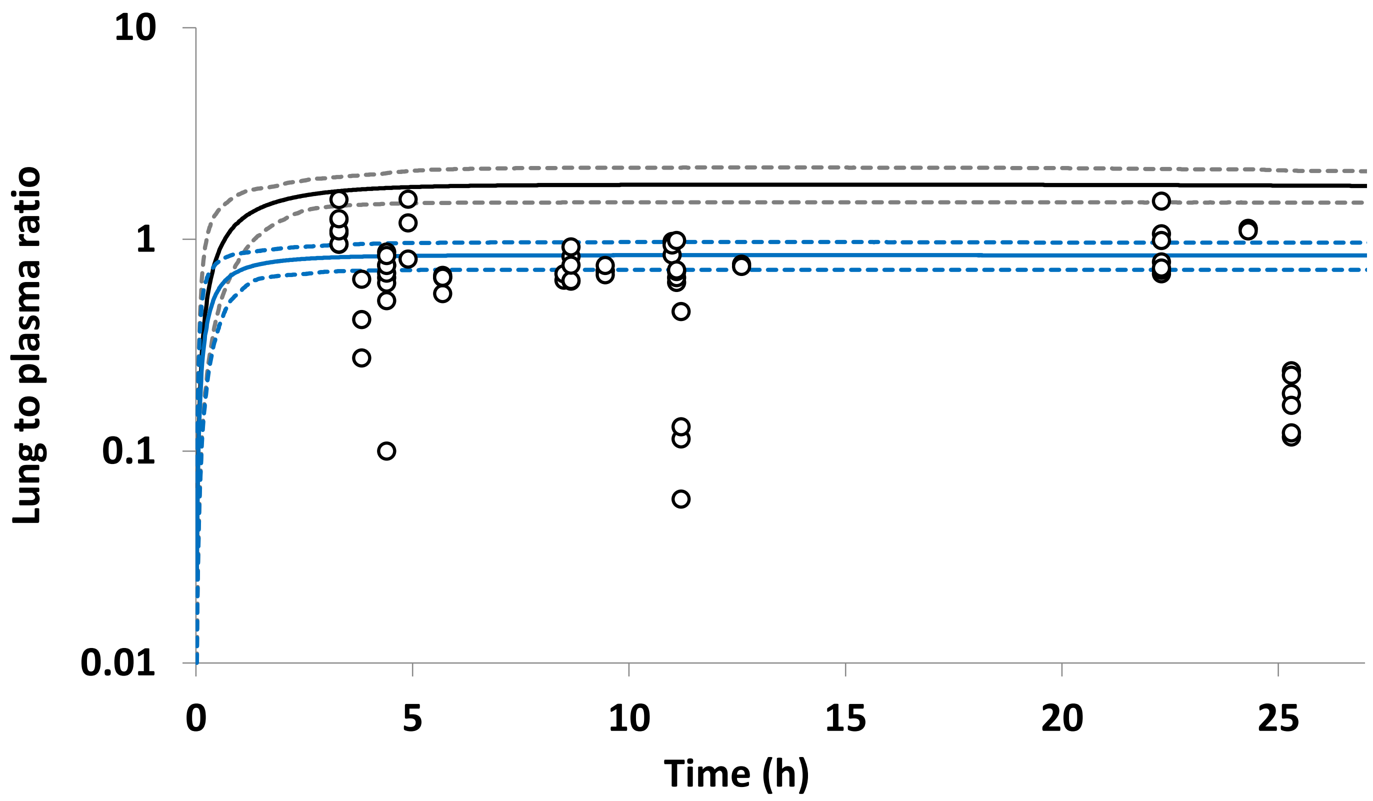

Figure 5. Simulated versus observed lung tissue_mass:plasma concentration ratio over time for rifampicin following a single dose of 600 mg, administered to TB patients. Observed (data points) data from Prideaux *et al.*, 2015[11]. The solid lines represent simulations using rifampicin lung fu_{mass} equal to either the predicted value of 0.058 (black) or the optimised value of 0.116 (blue). The short dashed lines represent the 5th and 95th percentile of the total virtual population of 10 trials of 15 healthy volunteers, 23 - 50 years, 33% female[11]. Simulated data are from the right lung (RL) compartment.
