# Supplementary material for: Development of physiologically‐based pharmacokinetic models for standard of care and newer tuberculosis drugs
Source: CPT Pharmacometrics Syst Pharmacol. 2021 Oct 8;10(11):1382–95. doi: 10.1002/psp4.12707 (PMC8592506; doi:10.1002/psp4.12707)
Supplement: Supplementary file 3 — Table S1–S5 [file PSP4-10-1382-s002.docx]

# Supplementary Material Methods

## **PBPK Model Input Parameters**

**Table S1. Physicochemical and Absorption PBPK Model Input Parameters**

|  | **Compound Type** | **pKa** ^a^ | **Log*P*_o:w_** | **BP** | **f_u,p_** | **Absorption Model** ^b^ | **References** |
| --- | --- | --- | --- | --- | --- | --- | --- |
| **Bedaquiline** | Monoprotic base | 9.1 *^c^* | 1 *^e^* | 1 *^c^* | 0.0005 *^c^* | First Order | ^1, 2, 3^ |
| ***N*-desmethyl bedaquiline** | Monoprotic base | 8.35 *^c^* | 6.51 *^d^* | 1 *^c^* | 0.0005 | - | ^1, 2^ |
| **Clofazimine** | Monoprotic base | 8.51 *^c^* | 7.44 *^d^* | 0.55 *^c^* | 0.001 *^d^* | First Order | ^2, 4^ ^5^ |
| **Cycloserine** | Ampholyte | 4.5, 7.5 *^c^* | -1.92 *^d^* | 0.55 | 0.75 *^f^* | First Order | ^6, 7^ |
| **Ethambutol** | Diprotic Base | 9.55, 6.5 *^c^* | 0.059 *^c^* | 1.3 *^c^* | 0.75 *^c^* | First Order | ^8, 9, 10^ |
| **Ethionamide** | Monoprotic Base | 5 *^d^* | 1.22 *^d^* | 1.17 *^f^* | 0.7 *^c^* | ADAM | ^2, 11, 12^ |
| **Isoniazid** | Monoprotic Base | 1.82 *^c^* | -0.7 *^c^* | 0.825 | 0.95 *^c^* | First Order | ^10, 13, 14, 15, 16^ |
| **Kanamycin** | Monoprotic Base | 9.5 *^d^* | -2.58 *^d^* | 0.644 *^f^* | 0.99 *^c^* | im (venous blood,  fa 1, ka 2 *^h^*) | ^2, 16, 17^ |
| **Linezolid** | Monoprotic Base | 1.8 *^c^* | 0.9 *^c^* | 1 | 0.69 *^c^* | First Order | ^18, 19^ |
| **Pyrazinamide** | Neutral | 13.9, 0.87 *^d^* | -0.95 *^d^* | 0.63 *^f^* | 0.9 | First Order | ^2, 10, 20, 21^ |
| **Rifampicin** | Ampholyte | 1.7, 7.9 *^c^* | 4.01 *^c^* | 0.9 *^c^* | 0.116 *^c^* | First Order | ^10, 22, 23, 24, 25, 26, 27^ |
| **Rifapentine** | Ampholyte | 4.97, 7.67 *^d^* | 4 *^d^* | 1 | 0.02 *^c^* | ADAM | ^21, 28, 29, 30^ |

^a^ acidic, basic if two values

*^b^* ADAM represents Advanced Dissolution Absorption and Metabolism

*^c^* Experimental value

*^d^* Predicted using external prediction tool

*^e^* Input as Log P = 1 to improve simulation speed (Log P = 7.89 predicted using Simcyp V14 and experimental Log D_O:W_ = 5.14 ^1^)

*^f^* Predicted using Simcyp V16

*^g^* Predicted using Simcyp V15

*^h^* Optimised against clinical data

**Table S2. Distribution and Elimination PBPK Model Input Parameters in Final PBPK models**

|  | **Kp Scalar** | **V_SS_ (L/kg)** | **fu_mass_** | **Henry’s Constant**  **(Pa^-3^/mole) *^d^*** | **Lung effective permeability**  **(10^-4^ cm/s)** | **Elimination** | **CL_R_ (L/h)** | **References** |
| --- | --- | --- | --- | --- | --- | --- | --- | --- |
| **Bedaquiline** | 0.04 | 9.36 *^a,b^* (Additional  organ Kp 838.9) | 0.00030 *^f^* | 4.47 E-13 | 0.48 *^e^* | CL_int_ 122 µl/min/mg HLM, additional CL 2.23 L/h *^c^* | 0 | ^1, 3, 31^ |
| ***N*-desmethyl bedaquiline** | 1.2 | 18.03 *^b^* | 0.00034 *^f^* | 2.03 E-13 | 8.42 *^e^* | CLpo 3.49 L/h *^c^* | 0 | ^1, 31^ |
| **Clofazimine** | 0.27 | 47.8 *^b^* | 0.00019 *^f^* | 3.64 E-06 | 25.127 *^e^* | CL_int_ 1160 µl/min/mg HLM *^c^* | 0 | ^4^ |
| **Cycloserine** | 1 | 0.31 *^f^* | 0.9999 *^f^* | 6.58 E-07 | 0.001 *^b^* (initial prediction was 1.46 *^e^*) | CL_int_ 0.08 µl/min/mg HLM *^c^* | 0.56 | ^32, 33^ |
| **Ethambutol** | 0.2 | 1.23 *^b^* | 0.359 *^f^* | 3.65 E-14 | 0.479 *^e^* | Additional systemic CL 4.35 L/h *^c^* | 25.55 | ^9, 10, 34, 35, 36^ |
| **Ethionamide** | 1 | 0.778 *^f^* | 0.824 *^f^* | 7.16 E-15 | 0.067 *^e^* | CL_int_ 13.5 µl/min/mg HLM, Lung CL 6.15 *^g^* | 0.043 | ^11, 37^ |
| **Isoniazid** | 1 | 0.50 *^f^* | 0.984 *^f^* | 1.21 E-14 | 0.21 *^h^* | CL_int_ 3.125 µl/min/mg cytosol *^c^* | 2.76 | ^10, 38, 39^ |
| **Kanamycin** | 0.2 | 0.236 *^b^* | 0.999 *^f^* | 2.95 E-33 | 0.00617 *^e^* | 0 | 4.74 | ^17, 40^ |
| **Linezolid** | 1 | 0.56 *^f^* | 0.90 *^f^* | 1.78 E-09 | 0.06 *^e^* | CL_int_ 2.03 µl/min/mg HLM *^c^* | 2.2 | ^41^ |
| **Pyrazinamide** | 1 | 0.46 *^f^* | 0.985 *^f^* | 1.20 E-12 | 0.0138 *^e^* | CL_int_ 0.5 µl/min/mg cytosol *^c^* | 0.11 | ^10, 42^ ^43, 44^ |
| **Rifampicin** | 0.1 | 0.43 *^b^* | 0.058 *^f^* | 1 E-37 | 36000 *^g^* | CL_int_ 17.95 µl/min/mg HLM *^c^* | 1.26 | ^10, 45, 46, 47, 48^ |
| **Rifapentine** | 0.1 | 0.58 *^b^* | 0.02 *^i^* | 1 E-34 | 170.3 *^h^* | CL_int_ 0.33 µl/min/pmol CYP2E1 (AADAC) *^c^* | 0.016 | ^30^,^49^ |

A Full PBPK model was used for all compounds with Method 2^50, 51, 52^ for distribution.

The Kp Scalar is a scalar applied to all predicted tissue Kp values

*^a^* Additional perfusion-limited organ incorporated with identical physiology as adipose and Kp optimized against healthy volunteer plasma concentration data^53^

*^b^* optimized against clinical data

*^c^* in vivo clearance value from clinical study or retrograde CLint using in vivo clearance value

*^d^* Predicted using EPI Suite ^54^

*^e^* Predicted using Log D and Hydrogen Bond Donor QSAR model^10^. Log D calculated at pH 6.5 from Log P using Simcyp V16. Corrected for fraction unionised at pH 7.4

*^f^* Predicted using Simcyp V16

*^g^* Experimental value

*^h^* Predicted using Caco-2 Papp QSAR model^10^. Corrected for fraction unionised at pH 7.4

*^i^* Predicted using Simcyp V16 (0.0018) and corrected for optimized Kp Scalar value (0.1)

**Table S3. Clinical study demography and PBPK simulation design**

| **Compound (dose)** | | | **Number of subjects** | | **Observed population** | **Virtual Population** | | | **Proportion females** | | **Age range** | | **Reference** | | |
| --- | --- | --- | --- | --- | --- | --- | --- | --- | --- | --- | --- | --- | --- | --- | --- |
| **Dheda *et al.,* 2018**  See Figure 3 | | | 12 | | TB patients (SA) | SA TB | | | 0.67 | | 23 - 50 | | ^32^ | | |
| **Prideaux *et al.,* 2015**  See Figure 3 | | | 15 | | TB patients (S Korea) | Sim-NEC | | | 0.33 | | 23 – 59 | | ^56^ | | |
| **Bedaquiline** | | |  | |  |  | | |  | |  | |  | | |
| 50mg po qd *^a^* | | | 6 | | HV | Sim-HV | | | 0 | | 19 – 37 | | ^53^ | | |
| 150mg po qd *^a^* | | | 6 | | HV | Sim-HV | | | 0 | | 20 – 33 | | ^53^ | | |
| 400mg po qd *^a^* | | | 6 | | HV | Sim-HV | | | 0 | | 20 - 25 | | ^53^ | | |
| 10mg po sd *^b^* | | | 6 | | HV | Sim-HV | | | 0 | | 20 – 27 | | ^53^ | | |
| 30mg po sd *^b^* | | | 6 | | HV | Sim-HV | | | 0 | | 21 – 40 | | ^53^ | | |
| 100mg po sd *^b^* | | | 6 | | HV | Sim-HV | | | 0 | | 18 – 36 | | ^53^ | | |
| 300mg po sd *^b^* | | | 6 | | HV | Sim-HV | | | 0 | | 20 – 44 | | ^53^ | | |
| 450mg po sd *^b^* | | | 6 | | HV | Sim-HV | | | 0 | | 21 – 38 | | ^53^ | | |
| 700mg po sd *^b^* | | | 6 | | HV | Sim-HV | | | 0 | | 18 - 30 | | ^53^ | | |
| 400mg qd, 14 days (± Ketoconazole 400mg qd days 12-14) *^a^* | | | 12 | | HV | Sim-HV | | | 0 | | 20 - 50 | | ^57^ | | |
| 300mg sd day 7 (± Rifampicin 600mg qd, 7 days) *^b^* | | | 16 | | HV | Sim-HV | | | 0 | | 20 - 50 | | ^58^ | | |
| 400mg qd 2wks, 200mg tiw 21wks *^b^* | | | 17 | | TB (mixed) | SA TB | | | 0.31 | | 20 - 68 | | ^1^ | | |
| **Clofazimine** | | |  | |  |  | | |  | |  | |  | | |
| 50mg po sd *^a^* | | | 12 | | HV | Sim-HV | 0.25 | | | | 35 - 64 | | ^4^ | | |
| 100mg po sd *^a^* | | | 12 | | HV | Sim-NEC | 0.50 | | | | 45 - 70 | | ^4^ | | |
| 200mg po sd *^b^* | | | 3 | | HV | Sim-HV | | 0 | | | 30 - 60 | ^59^ | | |  |
| **Cycloserine** | | |  | |  |  | | | |  |  | |  | |  |
| 125–2250 mg per day (cycloserine) *^a^* | | | 56 | | TB patients Bangladesh/USA | Sim-HV | | | | 0.251 | 18 - 65 | | ^33^ | |  |
| **Ethambutol** | | |  | |  |  | | | |  |  | |  | |  |
| 25mg/kg po single | | | 14 | | HV | Sim-NEC | | | | 0.43 | 30 - 55 | | ^36^ | |  |
| 25mg iv inf (10mins) single *^b^* | | | 1 | | Leprosy | Sim-HV | | | | 0.5 | 20 - 50 | | ^60^ | |  |
| 250mg po single *^b^* | | | 40 | | HV | Sim-HV | | | | 0.075 | 18 - 53 | | ^61^ | |  |
| **Isoniazid** |  |  | |  |  |  | | | | | | | |  |  |
| 300mg po single | | | 14 | | HV | Sim-NEC | | | | 0.43 | 30 - 55 | | ^62^ | |  |
| **Kanamycin** | | |  | |  |  | | | |  |  | |  | |  |
| 500mg im single *^b^* | | | 24 | | HV | Sim-HV | | | | 0 | 21 - 48 | | ^17^ | |  |
| **Linezolid** | | |  | |  |  | | | |  |  | |  | |  |
| 375mg iv inf (30mins) or po single | | | 12 | | HV | Sim-HV | | | | 0.417 | 25 - 53 | | ^63^ | |  |
| 625mg po bid | | | 6 | | HV | Sim-HV | | | | 0 | 22 - 48 | | ^41^ | |  |
| **Pyrazinamide** | | |  | |  |  | | | |  |  | |  | |  |
| 1500mg po single | | | 14 | | HV | Sim-HV | | | | 0 | 20 - 50 | | ^43^ | |  |
| **Rifampicin** | | |  | |  |  | | | |  |  | |  | |  |
| 600mg po sid | | | 6 | | HV | Sim-HV | | | | 0.5 | 20 - 50 | | ^64^ | |  |
| **Rifapentine** | | |  | |  |  | | | |  |  | |  | |  |
| 900mg single po (fasted) | | | 16 | | HV | Sim-HV | | | | 0.353 | 19 - 55 | | ^49^ | |  |
| 900mg single po (fed) | | | 15 | | HV | Sim-HV | | | | 0.2 | 24 - 64 | | ^65^ | |  |

*^a^* used for model development

*^b^* used for model verification

Sim-HV represents the V16 Simcyp library Sim-Healthy Volunteer population, Sim-NEC represents the V16 Simcyp library Sim-North European Caucasian population. SA TB is a virtual South African TB population with associated specific demographic and physiological parameters for this population, described in methods and supplementary material

**Table S4. Comparison of predicted and observed plasma AUC, Cmax and tmax values for 10 TB drugs administered to healthy volunteers**

| **Compound (dose)** | **Cmax (mg/L)** | | | **tmax (h)** | | | **AUC (mg/l.h)** | | | | **References** |
| --- | --- | --- | --- | --- | --- | --- | --- | --- | --- | --- | --- |
|  | **Observed** | **Predicted** | **Pred/**  **Obs** | **Observed** | **Predicted** | **Pred/**  **Obs** | **AUC time (h)** | **Observed** | **Predicted** | **Pred/**  **Obs** |  |
| **Bedaquiline** |  |  |  |  |  |  |  |  |  |  |  |
| 50mg po sid (day 1) *^c^* | 0.43 | 0.33 | 0.77 | 5.3 | 6.05 | 1.14 | 0 - 24 | 3.99 | 5.23 | 1.31 | ^53^ |
| 150mg po sid (day 1) *^c^* | 1.13 | 1.01 | 0.89 | 5.0 | 6.05 | 1.21 | 0 – 24 | 9.92 | 15.82 | 1.60 | ^53^ |
| 400mg po sid (day 1) *^c^* | 3.01 | 2.71 | 0.90 | 3.7 | 5.88 | 1.59 | 0 – 24 | 27.21 | 42.35 | 1.56 | ^53^ |
| 50mg po sid (day 14) *^c^* | 0.59 | 0.50 | 0.84 | 5.2 | 5.68 | 1.09 | 0 – 24 | 7.91 | 86.80 | 1.10 | ^53^ |
| 150mg po sid (day 14) *^c^* | 1.97 | 1.52 | 0.77 | 5.0 | 5.66 | 1.13 | 0 – 24 | 24.27 | 26.69 | 1.10 | ^53^ |
| 400mg po sid (day 14) *^c^* | 4.30 | 4.20 | 0.98 | 5.0 | 5.60 | 1.12 | 0 – 24 | 51.53 | 73.66 | 1.43 | ^53^ |
| 10mg po single *^d^* | 0.069 | 0.068 | 0.99 | 6.3 | 6.37 | 1.01 | 0 – infinity | 1.70 | 2.58 | 1.52 | ^53^ |
| 30mg po single *^d^* | 0.28 | 0.20 | 0.73 | 5.0 | 6.46 | 1.29 | 0 – infinity | 6.05 | 7.53 | 1.25 | ^53^ |
| 100mg po single *^d^* | 0.85 | 0.68 | 0.79 | 4.7 | 6.44 | 1.37 | 0 – infinity | 18.13 | 25.38 | 1.40 | ^53^ |
| 300mg po single *^d^* | 2.55 | 2.03 | 0.80 | 4.2 | 6.46 | 1.54 | 0 – infinity | 53.11 | 43.69 | 0.82 | ^53^ |
| 450mg po single *^d^* | 3.76 | 3.05 | 0.81 | 4.5 | 6.47 | 1.44 | 0 – infinity | 79.18 | 65.44 | 0.83 | ^53^ |
| 700mg po single *^d^* | 6.75 | 4.78 | 0.71 | 5.2 | 6.38 | 1.23 | 0 – infinity | 133.13 | 180.10 | 1.35 | ^53^ |
|  |  | **GMFE** | **0.83** |  | **GMFE** | **1.26** |  |  | **GMFE** | **1.24** |  |
|  |  | **Within 1.5-fold** | **100%** |  | **Within**  **1.5-fold** | **83%** |  |  | **Within**  **1.5-fold** | **75%** |  |
| ***N*-desmethyl bedaquiline** |  |  |  |  |  |  |  |  |  |  |  |
| 50mg po sid (day 1) *^c^* | 0.007 | 0.008 | 1.17 | 8.7 | 11.93 | 1.37 | 0 - 24 | 0.11 | 0.17 | 1.53 | ^53^ |
| 150mg po sid (day 1) *^c^* | 0.021 | 0.024 | 1.17 | 12.0 | 11.93 | 0.99 | 0 – 24 | 0.37 | 0.53 | 1.44 | ^53^ |
| 400mg po sid (day 1) *^c^* | 0.052 | 0.061 | 1.16 | 8.0 | 12.26 | 1.53 | 0 – 24 | 0.84 | 1.31 | 1.56 | ^53^ |
| 50mg po sid (day 14) *^c^* | 0.060 | 0.061 | 1.01 | 8.2 | 7.16 | 0.87 | 0 – 24 | 1.20 | 1.42 | 1.18 | ^53^ |
| 150mg po sid (day 14) *^c^* | 0.28 | 0.18 | 0.66 | 5.4 | 7.32 | 1.36 | 0 – 24 | 5.45 | 4.23 | 0.78 | ^53^ |
| 400mg po sid (day 14) *^c^* | 0.44 | 0.44 | 1.00 | 11.0 | 7.39 | 0.67 | 0 – 24 | 8.78 | 10.23 | 1.17 | ^53^ |
|  |  | **GMFE** | **1.03** |  | **GMFE** | **1.13** |  |  | **GMFE** | **1.24** |  |
|  |  | **Within 1.5-fold** | **100%** |  | **Within**  **1.5-fold** | **83%** |  |  | **Within**  **1.5-fold** | **67%** |  |
| **Clofazimine** |  |  |  |  |  |  |  |  |  |  |  |
| 50mg single po *^c^* | 71.8 | 73.5 | 1.02 | 8 | 6.21 | 0.78 | 0 - 24 | 1084 | 842 | 0.78 | ^4^ |
| 100mg single po *^c^* | 131 | 161 | 1.23 | 6 | 6.14 | 1.02 | 0 - 24 | 1856 | 1823 | 0.98 | ^4^ |
| 200mg single po *^d^* | 469 | 601 | 1.28 | 12 | 6.33 | 0.53 | 0 - 264 | 18000 | 27010 | 1.50 | ^59^ |
|  |  | GMFE | 1.17 |  | GMFE | 0.65 |  |  | GMFE | 1.05 |  |
|  |  | **Within 1.5-fold** | **100%** |  | **Within**  **1.5-fold** | **100%** |  |  | **Within**  **1.5-fold** | **100%** |  |
| **Ethambutol** |  |  |  |  |  |  |  |  |  |  |  |
| 25mg/kg single po | 4.55 | 3.69 | 0.81 | 2.48 | 3.15 | 1.27 | 0 - 48 | 28.1 | 38.35 | 1.37 | ^36^ |
| **Ethionamide** |  |  |  |  |  |  |  |  |  |  |  |
| 25mg single iv  inf (10min) *^d^* |  |  |  | - | - | - | 0 - infinity | 0.917 | 0.981 | 0.94 | ^60^ |
| 250mg single po *^d^* | 2.16 | 2.05 | 0.95 | 1.0 | 0.91 | 0.91 | 0 - infinity | 7.67 | 6.81 | 0.89 | ^61^ |
|  |  |  |  |  |  |  |  |  | **GMFE** | **0.91** |  |
|  |  |  |  |  |  |  |  |  | **Within**  **1.5-fold** | **100%** |  |
| **Isoniazid** |  |  |  |  |  |  |  |  |  |  |  |
| 300mg single po *^d^* | 5.88 | 6.57 | 1.12 | 0.91 | 0.33 | 0.37 | 0 - 48 | 18.5 | 27.52 | 1.49 | ^62^ |
| **Kanamycin** |  |  |  |  |  |  |  |  |  |  |  |
| 500mg single im *^d^* | 20.6 | 19.4 | 0.94 | 1.0 | 0.94 | 0.94 | 0 - 12 | 90 | 95.39 | 1.06 | ^17^ |
| **Linezolid** |  |  |  |  |  |  |  |  |  |  |  |
| 375mg single iv inf (30mins) *^c^* | 10.8 | 12.94 | 1.20 | - | - | - | 0 – infinity | 50.3 | 55.3 | 1.10 | ^63^ |
| 375mg single po *^d^* | 7.6 | 5.69 | 0.75 | 1.5 | 1.58 | 1.05 | 0 - infinity | 51.7 | 48.3 | 0.93 | ^63^ |
| 625mg po bid *^d^* | 18.75 | 11.12 | 0.59 | 2.12 | 1.47 | 0.69 | 0 - 12 | 147 | 81.6 | 0.56 | ^41^ |
|  |  | **GMFE** | **0.96** |  | **GMFE** |  |  |  | **GMFE** | **1.01** |  |
|  |  | **Within 1.5-fold** | **100%** |  | **Within 1.5-fold** | **100%** |  |  | **Within 1.5-fold** | **100%** |  |
| **Pyrazinamide** |  |  |  |  |  |  |  |  |  |  |  |
| 1500mg single po | 26.04 | 21.32 | 0.82 | 1.77 | 2.38 | 1.35 | 0 - 24 | 295.28 | 267.5 | 0.91 | ^43^ |
| **Rifampicin** |  |  |  |  |  |  |  |  |  |  |  |
| 300mg bid po *^d^* | 19.11 | 10.47 | 0.55 | 1 | 1.53 | 1.53 | 312 - 324 | 20.38 | 32.04 | 1.57 | ^64^ |
| 600mg sid po *^d^* | 15.00 | 11.25 | 0.75 | 2.0 | 1.53 | 0.77 | 312 - 336 | 70.35 | 72.03 | 1.02 | ^64^ |
| **Rifapentine** |  |  |  |  |  |  |  |  |  |  |  |
| 900mg single po (fasted) *^e,d^* | 18.4 | 13.1 | 0.71 | 4 | 4.7 | 1.18 | 0 – 72 | 560 | 402 | 0.72 | ^66^ |
| 900mg single po (fed) *^d^* | 21.9 | 18.5 | 0.85 | 5.1 | 4.5 | 0.88 | 0 - 48 | 513 | 516 | 1.01 | ^65^ |

Values are arithmetic mean except where noted otherwise

Cycloserine model is for multiple dosing only (not single). No multiple dosing clinical data for healthy volunteers

*^a^* median

^b^ To match the clinical study the frequency of EM and PM individuals was set to 0.286 : 0.714

*^c^* used to develop the PBPK model

*^d^* used to verify the PBPK model

*^e^* geometric mean

## **Compound Specific PBPK Model Assumptions**

### **Isoniazid**

Isoniazid is metabolized by the polymorphically expressed *N*-acetyltransferase 2 enzyme (NAT-2) ^67^. To account for known population variability in NAT-2 metabolism for extensive (EM), poor (PM) and intermediate (IM) metabolisers, relative NAT-2 efficiency (mean (CV) = 1 (30%), 0.29 (30%) and 0.61 (30%), respectively) and South African TB population frequency (mean = 0.236, 0.425 and 0.339, respectively) were input based on meta-analysis ^67, 68^ (described in detail in Supplementary Material). For simulation of a Korean clinical study, relative NAT-2 efficiency (mean (CV) = 1 (30%), 0.29 (30%) and 0.61 (30%), respectively) and population frequency (mean = 0.734, 0.133 and 0.133, respectively) were assigned to match the subjects in the specific clinical study ^56, 69^.

### **Bedaquiline**

To accurately describe the tri-phasic profile of bedaquiline after oral administration, an additional organ compartment was included in the PBPK model. The fraction metabolised by CYP 3A4 in the bedaquiline file was optimised using data from a study with ketoconazole (a strong CYP 3A4 inhibitor) and independently verified using a drug interaction study with rifampicin.

### **Ethionamide**

Ethionamide is extensively metabolised by Flavin-containing monooxygenase isoform 3 in the body. In the present model, *in vitro* measured intrinsic clearance (CL_int_) values in pooled human liver and lung microsomes ^11^ were incorporated and scaled using reported liver and lung scaling factors ^70, 71, 72^.

### **Rifapentine**

The PBPK model for rifapentine used a mechanistic absorption model ^73^ to allow the changes in pharmacokinetics in fed and fasted states to be simulated mechanistically. The hepatic CL_int_ was calculated using a retrograde approach based on a meta-analysis of oral clearance values of rifapentine in the fed state with the assumption that under these conditions the absorption of rifapentine is complete. Induction of CYP 3A4 by rifapentine was incorporated into the PBPK model using *in vitro* CYP 3A4 mRNA data (*Ind*_max_ = 28.7; *IndC*_50_= 2.52 µM), which were calibrated against rifampicin data measured in the same study (*Ind*_max_ = 32.2; *IndC*_50_ = 1.07 µM) ^74^. The final calibrated values used for rifapentine CYP 3A4 induction were *Ind*_max_ = 14.3 and *IndC*_50_= 0.75 µM. To enable the simulation of auto-induction of Rifapentine metabolism observed on multiple dosing, the metabolism parameters were entered into the Rifapentine model using a surrogate enzyme (CYP 2E1) as arylacetamide deacetylase (AADAC) because of software limitations.

# Supplementary Material Results

## **Simulations of drug distribution to additional lung compartments (epithelial lining fluid, alveaolar cells or tissue) of TB patients**

For some compounds, additional studies that measured drug concentrations in lung tissue, alveolar cells and/or epithelial lining fluid were identified and simulated data were compared to the observed clinical data (Figure 4 and Table S1).

For ethambutol, Conte et al reported the concentration of ethambutol in plasma (2.1 ± 0.8 mg/L), ELF (2.2 ± 1.1 mg/L) and alveolar macrophages (range 45 ± 16 mg/L to 82 ± 39 mg/L) of different sub-groups of healthy volunteers and AIDS patients using bronchial lavage (4 hours after multiple dosing of ethambutol) ^75^. Simulations were in line with observed at the same time point for plasma (2.2 ± 0.8 mg/L) and ELF (1.7 ± 0.7 mg/L) but were significantly lower than the measured value in the lung tissue (3.4 +/- 1.4 mg/L). Sensitivity analyses showed that decreasing the lung mass pH or increasing binding in the lung mass (decreasing fu_mass_) increased simulated lung mass to be within range of reported values (Figure 6A). The dibasic nature of ethambutol makes it very sensitive to local pH.

For ethionamide, the plasma to ELF ratio was measured in the right middle lung lobe at 4 hour after multiple oral doses of 250 mg. The reported mean ELF to plasma concentration ratio was 5.00 in male and 6.89 in female, respectively ^56^. The initially developed PBPK model for ethionamide under-predicted the mean ELF to plasma concentration ratio by approximately 10-fold. After accounting for P-glycoprotein activity by scaling available *in vitro* data ^11^, simulated ELF to plasma concentration ratio increased to 6.41 +/- 0.75 in male and 6.43 +/- 0.8 in female, in line with the clinically observed data ^56^. In a different clinical study, lung concentration was measured as 0.148 mg/L in one individual at 5 hours after dosing with ethionamide for multiple doses ^76^, (below the reported LLOQ of 0.16 mg/L). The range in simulated lung concentration for different individuals (0.08 – 2.24 mg/L, mean 0.82 mg/L) at the same time point covered the observed data (Figure 3).

For Linezolid the reported ELF:plasma ratios in different clinical studies ^77, 78, 79, 80^ vary markedly and the simulated results are in line with the results reported in 2 of the 4 clinical studies. Concentrations of linezolid in alveolar cells and/or bronchial biopsies was measured in 2 of the 4 clinical studies ^79, 80^. The simulated concentrations of linezolid in the lung mass compartment showed reasonable agreement with the measured concentrations, particularly when the variability in the clinically measured studies is considered (Figure S3). Sensitivity analysis showed that increasing the numerical value of the fraction unbound in the lung (fu_mass_) over a 3-fold range gave simulated concentrations more in line with the reported concentrations in the study by Conte *et al.,* ^79^ (data not shown).

In addition, pyrazinamide lung cell and plasma concentrations were measured in a clinical study reported by Conte *et al*., ^81^. In that study the concentrations in plasma and lung cells 4 hours after the last dose of pyrazinamide were 21.1 +/- 6.8 mg/L and 17.4 +/- 16.9, respectively. The lung cell/plasma ratio was 0.83 +/- 0.7. In the simulated study the pyrazinamide concentrations in the lung and plasma were 17 +/- 6.9 mg/L and 18.5 +/- 7.5, respectively with a mean ratio of 0.92.

The intrapulmonary pharmacokinetics of rifapentine have been described in a study by Conte *et al.,* ^82^. The initial PBPK model developed for Rifapentine under-predicted the ELF:plasma ratio reported in the clinical study by a factor of ~5-6. Sensitivity analysis on some of the uncertain parameters within the model (ELF fraction unbound (fu_ELF_), and ELF pH with compound acid pKa) resulted in simulated results that were in line with the clinical data. The study by Conte *et al.,* also provides data on the concentrations within cells collected during the alveolar lavage procedure. With the assumption that these concentrations are reflective of the concentration seen in lung tissue the reported concentrations were compared to the simulated concentrations in the lung mass with a study design (dose of rifapentine, age and number of subjects) matching those reported in the clinical study. Although the reported mean cell concentrations were within the range of simulated values at all time points there was a tendency for the simulations to over predict the concentrations compared to the measured concentrations. Sensitivity analysis showed that increasing the numerical value of fu_mass_ (over the range 0.02 to 0.06) improved simulation of the clinically measured concentrations (Figure 6C).

**Table S5 Summary of simulated lung concentrations using the PBPK models**

| **Compound** | **Prediction accuracy of initial PBPK model for lung concentrations or lung:plasma ratio** | **Sensitivity Analyses** | |
| --- | --- | --- | --- |
|  |  | **Parameters varied** | **Outcome** |
| Clofazimine* | Reasonably recovers observed data |  |  |
| Cycloserine* | Over-predicted | ELF pH  Lung permeability | Limited sensitivity |
| Ethionamide | Under-predicted ELF concentration |  | ELF concentrations recovered when P-gp accounted for |
| Ethambutol | Reasonably recovers observed data |  |  |
| Isoniazid | Reasonably recovers observed data |  |  |
| Kanamycin | Reasonably recovers observed data |  |  |
| Linezolid | Reasonably recovers ELF:plasma ratio in 2/4 clinical studies.  Reasonably recovers tissue:plasma ratio in 2/2 clinical studies | ELF pH  Lung fu_mass_ | Limited sensitivity in physiological range  Tissue concentrations decreased as lung fu_mass_ is numerically increased. |
| Pyrazinamide | Reasonably recovers observed data |  |  |
| Rifampicin | Reasonably recovers observed data |  |  |
| Rifapentine | Under-predicted ELF concentration (4-7-fold) | ELF fu  ELF pH  Compound acidic pKa | Varying these parameters, it is possible to simulate concentrations covering the range observed clinically. |

*caveat that the measured concentrations were below the lower limit of quantification of the assay.

1. Janssen. Investigator's Brochure (Edition 7). Janssen Infectious Diseases - Diagnostics BVBA for The International Union Against Tuberculosis and Lung Disease. Document No.: EDMS-ERI-10268920. . (2012)

2. ACD/Labs. ACD/Percepta 14.0.0, build 2254, Advanced Chemistry Development, Inc., Toronto, ON, Canada, w[ww.acdlabs.com.](https://s08sharepoint.certara.com/sites/RnD/TB_Critical%20Path%20Project/Compound%20Files/V19%20NWL16%20Cpath%20Compounds/ww.acdlabs.com.) (2012)

3. Svensson EM, Murray S, Karlsson MO, Dooley KE. Rifampicin and rifapentine significantly reduce concentrations of bedaquiline, a new anti-TB drug. *J Antimicrob Chemother* **70** 1106-1114. (2015)

4. Sangana R, Gu H, Chun DY, Einolf HJ. Evaluation of Clinical Drug Interaction Potential of Clofazimine Using Static and Dynamic Modeling Approaches. *Drug Metab Dispos* **46** 26-32. (2018)

5. Quigley JM, Fahelelbom KMS, Timoney RF, Corrigan OI. Temperature dependence and thermodynamics of partitioning of clofazimine analogues in the n-octanol/water system. *Int J Pharm* **58** 107-113. (1990)

6. McBain CJ, Kleckner NW, Wyrick S, Dingledine R. Structural requirements for activation of the glycine coagonist site of N-methyl-D-aspartate receptors expressed in Xenopus oocytes. *Mol Pharmacol* **36** 556-565. (1989)

7. Tetko IV*, et al.* Virtual computational chemistry laboratory--design and description. *J Comput Aided Mol Des* **19** 453-463. (2005)

8. Becker C*, et al.* Biowaiver monographs for immediate release solid oral dosage forms: ethambutol dihydrochloride. *J Pharm Sci* **97** 1350-1360. (2008)

9. Lee CS, Gambertoglio JG, Brater DC, Benet LZ. Kinetics of oral ethambutol in the normal subject. *Clin Pharmacol Ther* **22** 615-621. (1977)

10. Gaohua L*, et al.* Development of a Multicompartment Permeability-Limited Lung PBPK Model and Its Application in Predicting Pulmonary Pharmacokinetics of Antituberculosis Drugs. *CPT Pharmacometrics Syst Pharmacol* **4** 605-613. (2015)

11. Nguyen PTT*, et al.* Development of a Physiologically Based Pharmacokinetic Model of Ethionamide in the Pediatric Population by Integrating Flavin-Containing Monooxygenase 3 Maturational Changes Over Time. *J Clin Pharmacol* **58** 1347-1360. (2018)

12. Chemaxon. h[ttps://chemicalize.com/#/calculation](ttps://chemicalize.com/#/calculation ) . accessed on 01/12/2018. (2018)

13. Perrin DD. *Dissociation Constants of Organic Bases in Aqueous Solutions [Book]. IUPAC Chemical Data Series No. 12. Pure and Applied Chemistry*. London: Butterworths., 1965.

14. Hansch C, Leo A, Hoekman D. *Exploring QSAR. Hydrophobic, electronic, and steric constants. ACS Professional Reference Book*. ACS, Washington, 1995.

15. Herrera AM, Scott DO, Lunte CE. Microdialysis sampling for determination of plasma protein binding of drugs. *Pharm Res* **7** 1077-1081. (1990)

16. Lombardo F, Berellini G, Obach RS. Trend Analysis of a Database of Intravenous Pharmacokinetic Parameters in Humans for 1352 Drug Compounds. *Drug Metab Dispos* **46** 1466-1477. (2018)

17. Cabana BE, Taggart JG. Comparative pharmacokinetics of BB-K8 and kanamycin in dogs and humans. *Antimicrob Agents Chemother* **3** 478-483. (1973)

18. Pfizer. Zyvox Linezolid injection, tablets, oral suspension. FDA NDA 21-130/S-016/S-017, NDA 21-131/S-013/S-014, NDA 21-132/S-014/S-015. LAB-0139-17.0. (2008)

19. Hedaya MA, Thomas V, Abdel-Hamid ME, Kehinde EO, Phillips OA. Comparative Pharmacokinetic Study for Linezolid and Two Novel Antibacterial Oxazolidinone Derivatives in Rabbits: Can Differences in the Pharmacokinetic Properties Explain the Discrepancies between Their In Vivo and In Vitro Antibacterial Activities? *Pharmaceutics* **9**. (2017)

20. Becker C*, et al.* Biowaiver monographs for immediate release solid oral dosage forms: pyrazinamide. *J Pharm Sci* **97** 3709-3720. (2008)

21. DrugBank. w[ww.drugbank.com.](https://s08sharepoint.certara.com/sites/RnD/TB_Critical%20Path%20Project/Compound%20Files/V19%20NWL16%20Cpath%20Compounds/ww.drugbank.com.) (2015)

22. Pasqualucci CR, Vigevani A, Radaelli P, Maggi N. [Spectrophotometric analysis of rifampicin]. *Farmaco Prat* **24** 46-52. (1969)

23. Liu L*, et al.* Radiosynthesis and bioimaging of the tuberculosis chemotherapeutics isoniazid, rifampicin and pyrazinamide in baboons. *J Med Chem* **53** 2882-2891. (2010)

24. Stephen KW, McCrossan J, Mackenzie D, Macfarlane CB, Speirs CF. Factors determining the passage of drugs from blood into saliva. *Br J Clin Pharmacol* **9** 51-55. (1980)

25. Loos U*, et al.* Pharmacokinetics of oral and intravenous rifampicin during chronic administration. *Klin Wochenschr* **63** 1205-1211. (1985)

26. Boman G, Ringberger VA. Binding of rifampicin by human plasma proteins. *Eur J Clin Pharmacol* **7** 369-373. (1974)

27. Templeton IE, Houston JB, Galetin A. Predictive utility of in vitro rifampin induction data generated in fresh and cryopreserved human hepatocytes, Fa2N-4, and HepaRG cells. *Drug Metab Dispos* **39** 1921-1929. (2011)

28. Chembl. <https://www.ebi.ac.uk/chembl/compound/inspect/CHEMBL1660>. (2019)

29. Sanofi-Aventis. Prescribing Information. FDA. Priftin (rifapentine) tablets. Initial U.S. Approved: 1998 (2010)

30. Reith K*, et al.* Disposition and metabolism of 14C-rifapentine in healthy volunteers. *Drug Metab Dispos* **26** 732-738. (1998)

31. Janssen. Clinical Study Report. R207910-CDE-102. Document No.: EDMS-PSDB-4196530. Drug Evaluation Clinical Operations Department. (2005)

32. Dheda K*, et al.* Drug-Penetration Gradients Associated with Acquired Drug Resistance in Patients with Tuberculosis. *American journal of respiratory and critical care medicine* **198** 1208-1219. (2018)

33. Alghamdi WA*, et al.* Cycloserine Population Pharmacokinetics and Pharmacodynamics in Patients with Tuberculosis. *Antimicrob Agents Chemother*. (2019)

34. Lee CS, Brater DC, Gambertoglio JG, Benet LZ. Disposition kinetics of ethambutol in man. *J Pharmacokinet Biopharm* **8** 335-346. (1980)

35. Breda M*, et al.* Effect of rifabutin on ethambutol pharmacokinetics in healthy volunteers. *Pharmacol Res* **40** 351-356. (1999)

36. Peloquin CA*, et al.* Pharmacokinetics of ethambutol under fasting conditions, with food, and with antacids. *Antimicrob Agents Chemother* **43** 568-572. (1999)

37. Jenner PJ, Ellard GA, Gruer PJ, Aber VR. A comparison of the blood levels and urinary excretion of ethionamide and prothionamide in man. *J Antimicrob Chemother* **13** 267-277. (1984)

38. Ellard GA, Gammon PT. Pharmacokinetics of isoniazid metabolism in man. *J Pharmacokinet Biopharm* **4** 83-113. (1976)

39. McIlleron H*, et al.* Determinants of rifampin, isoniazid, pyrazinamide, and ethambutol pharmacokinetics in a cohort of tuberculosis patients. *Antimicrob Agents Chemother* **50** 1170-1177. (2006)

40. Obach RS, Lombardo F, Waters NJ. Trend analysis of a database of intravenous pharmacokinetic parameters in humans for 670 drug compounds. *Drug Metabolism and Disposition*. (2008)

41. Stalker DJ, Jungbluth GL, Hopkins NK, Batts DH. Pharmacokinetics and tolerance of single- and multiple-dose oral or intravenous linezolid, an oxazolidinone antibiotic, in healthy volunteers. *J Antimicrob Chemother* **51** 1239-1246. (2003)

42. Peloquin CA*, et al.* Pharmacokinetics of pyrazinamide under fasting conditions, with food, and with antacids. *Pharmacotherapy* **18** 1205-1211. (1998)

43. Agrawal S*, et al.* Assessment of bioequivalence of rifampicin, isoniazid and pyrazinamide in a four drug fixed dose combination with separate formulations at the same dose levels. *Int J Pharm* **233** 169-177. (2002)

44. Agrawal S*, et al.* Bioequivalence assessment of rifampicin, isoniazid and pyrazinamide in a fixed dose combination of rifampicin, isoniazid, pyrazinamide and ethambutol vs. separate formulations. *Int J Clin Pharmacol Ther* **40** 474-481. (2002)

45. Acocella G. Clinical pharmacokinetics of rifampicin. *Clin Pharmacokinet* **3** 108-127. (1978)

46. Peloquin CA, Namdar R, Singleton MD, Nix DE. Pharmacokinetics of rifampin under fasting conditions, with food, and with antacids. *Chest* **115** 12-18. (1999)

47. Polk RE*, et al.* Pharmacokinetic Interaction between amprenavir and rifabutin or rifampin in healthy males. *Antimicrob Agents Chemother* **45** 502-508. (2001)

48. Acocella G, Bonollo L, Mainardi M, Margaroli P, Tenconi LT. Serum and urine concentrations of rifampicin administered by intravenous infusion in man. *Arzneimittelforschung* **27** 1221-1226. (1977)

49. Sanofi Aventis. Rifapentine. FDA submission. Application number 21-024S008. (2009)

50. Rodgers T, Leahy D, Rowland M. Physiologically based pharmacokinetic modeling 1: predicting the tissue distribution of moderate-to-strong bases. *J Pharm Sci* **94** 1259-1276. (2005)

51. Rodgers T, Rowland M. Physiologically based pharmacokinetic modelling 2: predicting the tissue distribution of acids, very weak bases, neutrals and zwitterions. *J Pharm Sci* **95** 1238-1257. (2006)

52. Rodgers T, Rowland M. Mechanistic approaches to volume of distribution predictions: understanding the processes. *Pharmaceutical research* **24** 918-933. (2007)

53. Janssen Infectious Diseases - Diagnostics BVBA for The International Union Against Tuberculosis and Lung Disease. MAD Study 207910-CDE-102. Described in Investigator's Brochure. TMC207-TIDP13. (2012 September 18)

54. EPIsuite. Estimation Programs Interface Suite™ for Microsoft® Windows. United States Environmental Protection Agency, Washington, DC, USA. (2018)

55. Sarathy JP*, et al.* Prediction of drug penetration in tuberculosis lesions. *ACS infectious diseases* **2** 552-563. (2016)

56. Prideaux B*, et al.* The association between sterilizing activity and drug distribution into tuberculosis lesions. *Nature medicine* **21** 1223. (2015)

57. Janssen Infectious Diseases - Diagnostics BVBA for The International Union Against Tuberculosis and Lung Disease. Bedaquiline and Ketoconazole DDI study TMC207-C109. Described in Investigator's Brochure. TMC207-TIDP13. (2012 September 18)

58. Janssen Infectious Diseases - Diagnostics BVBA for The International Union Against Tuberculosis and Lung Disease. Bedaquiline and Rifampicin DDI study R207910BAC1003. Described in Investigator's Brochure. TMC207-TIDP13. (2012 September 18)

59. Schaad-Lanyi Z, Dieterle W, Dubois JP, Theobald W, Vischer W. Pharmacokinetics of clofazimine in healthy volunteers. *Int J Lepr Other Mycobact Dis* **55** 9-15. (1987)

60. Jenner PJ, Smith SE. Plasma levels of ethionamide and prothionamide in a volunteer following intravenous and oral dosages. *Lepr Rev* **58** 31-37. (1987)

61. Korth-Bradley JM, Mayer P, Mansfield D, Tucker H, Wu D. Comparative Bioavailability Study of Single-Dose Film-Coated and Sugar-Coated Ethionamide Tablets in Healthy Volunteers. *Clinical Therapeutics* **36** 982-987. (2014)

62. Peloquin CA, Namdar R, Dodge AA, Nix DE. Pharmacokinetics of isoniazid under fasting conditions, with food, and with antacids. *Int J Tuberc Lung Dis* **3** 703-710. (1999)

63. Welshman IR, Sisson TA, Jungbluth GL, Stalker DJ, Hopkins NK. Linezolid absolute bioavailability and the effect of food on oral bioavailability. *Biopharm Drug Dispos* **22** 91-97. (2001)

64. Acocella G, Pagani V, Marchetti M, Baroni G, Nicolis F. Kinetic studies on rifampicin. *Chemotherapy* **16** 356-370. (1971)

65. Dooley K*, et al.* Repeated administration of high-dose intermittent rifapentine reduces rifapentine and moxifloxacin plasma concentrations. *Antimicrob Agents Chemother* **52** 4037-4042. (2008)

66. Sanofi-Aventis. Rifapentine. FDA Clinical Pharmacology Review. NDA 21,024. Reference ID: 3649702. Page 64. (2014)

67. Weber WW, Hein DW. Clinical pharmacokinetics of isoniazid. *Clin Pharmacokinet* **4** 401-422. (1979)

68. Werely C. Pharmacogenetics of Arylamine N-acetyltransferase genes in South African populations. PhD thesis, Stellenbosch University, 2012.

69. Strydom N*, et al.* Tuberculosis drugs' distribution and emergence of resistance in patient's lung lesions: A mechanistic model and tool for regimen and dose optimization. *PLoS Med* **16** e1002773. (2019)

70. Prough RA, Sipal Z, Jakobsson SW. Metabolism of benzo(a)pyrene by human lung microsomal fractions. *Life Sci* **21** 1629-1635. (1977)

71. Valentin J. *ICRP Publciation 89: Basic anatomical and physiologcial data for use in radiological protection: reference values*. Pergamon, 2002.

72. Cubitt HE, Yeo KR, Howgate EM, Rostami-Hodjegan A, Barter ZE. Sources of interindividual variability in IVIVE of clearance: an investigation into the prediction of benzodiazepine clearance using a mechanistic population-based pharmacokinetic model. *Xenobiotica* **41** 623-638. (2011)

73. Jamei M*, et al.* Population-based mechanistic prediction of oral drug absorption. *AAPS J* **11** 225-237. (2009)

74. Almond LM*, et al.* Prediction of Drug-Drug Interactions Arising from CYP3A induction Using a Physiologically Based Dynamic Model. *Drug Metab Dispos* **44** 821-832. (2016)

75. Conte JE, Jr., Golden JA, Kipps J, Lin ET, Zurlinden E. Effects of AIDS and gender on steady-state plasma and intrapulmonary ethambutol concentrations. *Antimicrob Agents Chemother* **45** 2891-2896. (2001)

76. Dheda K*, et al.* Drug-Penetration Gradients Associated with Acquired Drug Resistance in Patients with Tuberculosis. *Am J Respir Crit Care Med* **198** 1208-1219. (2018)

77. Boselli E*, et al.* Alveolar diffusion and pharmacokinetics of linezolid administered in continuous infusion to critically ill patients with ventilator-associated pneumonia. *J Antimicrob Chemother* **67** 1207-1210. (2012)

78. Boselli E*, et al.* Pharmacokinetics and intrapulmonary concentrations of linezolid administered to critically ill patients with ventilator-associated pneumonia. *Crit Care Med* **33** 1529-1533. (2005)

79. Conte JE, Jr., Golden JA, Kipps J, Zurlinden E. Intrapulmonary pharmacokinetics of linezolid. *Antimicrob Agents Chemother* **46** 1475-1480. (2002)

80. Honeybourne D, Tobin C, Jevons G, Andrews J, Wise R. Intrapulmonary penetration of linezolid. *J Antimicrob Chemother* **51** 1431-1434. (2003)

81. Conte JE, Jr., Golden JA, Duncan S, McKenna E, Zurlinden E. Intrapulmonary concentrations of pyrazinamide. *Antimicrob Agents Chemother* **43** 1329-1333. (1999)

82. Conte JE, Jr.*, et al.* Single-dose intrapulmonary pharmacokinetics of rifapentine in normal subjects. *Antimicrob Agents Chemother* **44** 985-990. (2000)
